# Role of *N*–Oxide Moieties in Tuning Supramolecular Gel-State Properties

**DOI:** 10.3390/gels6040041

**Published:** 2020-11-20

**Authors:** Dipankar Ghosh, Ragnar Bjornsson, Krishna K. Damodaran

**Affiliations:** 1 Department of Chemistry, Science Institute, University of Iceland, Dunhagi 3, 107 Reykjavík, Iceland; dig11@hi.is; 2 Department of Inorganic Spectroscopy, Max–Planck–Institut für Chemische Energiekonversion, Campus de Stiftstrasse 34–36, 45470 Mülheim an der Ruhr, Germany; ragnar.bjornsson@gmail.com

**Keywords:** LMWGs, hydrogen bonding, pyridyl urea, *N*–oxide, structural modification, computational calculations, stimuli-responsive

## Abstract

The role of specific interactions in the self-assembly process of low molecular weight gelators (LMWGs) was studied by altering the nonbonding interactions responsible for gel formation via structural modification of the gelator/nongelator. This was achieved by modifying pyridyl moieties of bis(pyridyl) urea-based hydrogelator (**4–BPU**) and the isomer (**3–BPU**) to pyridyl *N*–oxide compounds (**L_1_** and **L_2_**, respectively). The modification of the functional groups resulted in the tuning of the gelation properties of the parent gelator, which induced/enhanced the gelation properties. The modified compounds displayed better mechanical and thermal stabilities and the introduction of the *N*–oxide moieties had a prominent effect on the morphologies of the gel network, which was evident from the scanning electron microscopy (SEM) images. The effect of various interactions due to the introduction of *N*–oxide moieties in the gel network formation was analyzed by comparing the solid-state interactions of the compounds using single crystal X-ray diffraction and computational studies, which were correlated with the enhanced gelation properties. This study shows the importance of specific nonbonding interactions and the spatial arrangement of the functional groups in the supramolecular gel network formation.

## 1. Introduction

Gels are fascinating semi-solid materials with unique physical characteristics which can retain their solid-like shape but display fluid-like properties on applying external stress [1]. Supramolecular gels based on low molecular weight gelators (LMWGs) [2,3,4,5,6,7] are materials with intriguing potential applications [5,6,8,9,10,11,12] such as sensing, catalysis, tissue engineering, drug delivery and as a media to control crystal growth. LMWGs offer several advantages over the conventional polymer gels due to their synthetic accessibility, tunable gel state property, inherent reversibility, and smart response towards external stimuli [3,4,5,13]. LMWGs are formed by the self-assembly of small molecules in the presence of solvents via various nonbonding interactions resulting in the formation of a three-dimensional superstructure with entrapped solvent molecules [2,3,4,5,6,14]. The principles of supramolecular chemistry helped researchers to understand the importance of nonbonding interactions and functional groups in designing LMWGs resulting in a myriad of LMWGs over the past decade with different functional groups [15,16,17,18,19,20,21,22,23,24]. However, the dynamic nature of the nonbonding interactions in LMWGs makes it difficult to predict the gel network formation, which is one of the reasons that the discovery of the majority of new LMWGs is serendipitous. Thus, the design of new LMWGs with predictable properties is still challenging because the geometry and spatial arrangement of the building blocks or functional groups dictate the properties and structure of LMWGs. The effect of various functional groups in the self-assembly process of LMWGs can be analyzed by comparing the gelation properties of LMWGs with similar structures, which will provide a better understanding of the self-assembly process.

An excellent strategy is to modify a well-known gelator comprising supramolecular synthons or hydrogen bonding moieties, which will enable us to compare the gelation and structural properties of the modified gelator with the parent gelator [25]. McNeil et al. have modified an azosulfonate gelator scaffold to develop a nitrite sensor based on LMWGs [26]. We have shown that the modification of the functional group of trimesic amide based gelator *N^1^,N^3^,N^5^*–tri(pyridin–3–yl)benzene–1,3,5–tricarboxamide [27] resulted in a *tris*–*N*–oxide compound with different gelation properties [28]. We have studied the importance of functional groups and the role of nonbonding interactions in tuning gelation properties of LMWGs by altering the intermolecular interactions. This was achieved by modifying the pyridyl groups of N–(4–pyridyl)isonicotinamide (4PINA) [29] to *N*–oxide groups, which resulted in two mono–*N*–oxides and a di–*N*–oxide compound [30]. The strong and unidirectional N—H∙∙∙N interactions of the parent gelator was disrupted by the modification of the pyridyl group and the effect of these interactions on the gelation properties was studied.

The tuning of gelation properties by functional group modification prompted us to explore this strategy to urea-based pyridyl compounds because urea and amide moieties are extensively used to generate LMWGs [19,31]. Generally, in urea-based LMWGs, the urea motifs self-assemble via complementary hydrogen bonding (N—H∙∙∙O=C) into one-dimensional arrays of hydrogen bonded chain to form α–tapes (fibrils) and the aggregation of these fibrils results in an entangled three-dimensional framework. The solid-state structural analysis of the gelators using single crystal X-ray diffraction (SCXRD) will provide information about the key interactions in the solid-state structure of LMWGs [29,32,33,34,35,36,37,38], which can be correlated to the interactions and the packing modes of these molecules in the gel state. The hydrogen-bonding synthons in these LMWGs are strongly influenced by the adjacent functional groups; for example, attaching pyridyl group to urea or amide moiety alters the intermolecular interactions resulting in an N—H∙∙∙N hydrogen bonding synthon involving the urea/amide group and pyridyl moiety [29,39]. Steed et al. reported that N—H∙∙∙N interactions also play an important role in the crystal structure of pyridyl–urea compounds [40,41]. These N—H∙∙∙N interactions can be replaced with N—H∙∙∙O interactions, which will enable us to study the effect of structural modification and the role of the resulting interaction in the supramolecular gelation of bis(pyridyl)urea compounds. We have selected *N,N’*–bis(3–pyridyl) urea (**3–BPU**) and *N,N’*–bis(4–pyridyl) urea (**4–BPU**) as the parent compounds [32] and the pyridyl nitrogen atoms of **3–BPU**/**4–BPU** were oxidized to corresponding *N*–oxides. The structural modification induced gelation or enhanced gelation properties were studied by analyzing the key nonbonding interactions of the parent and modified compounds.

## 2. Results and Discussion

### 2.1. Design and Synthesis

**Scheme 1:**
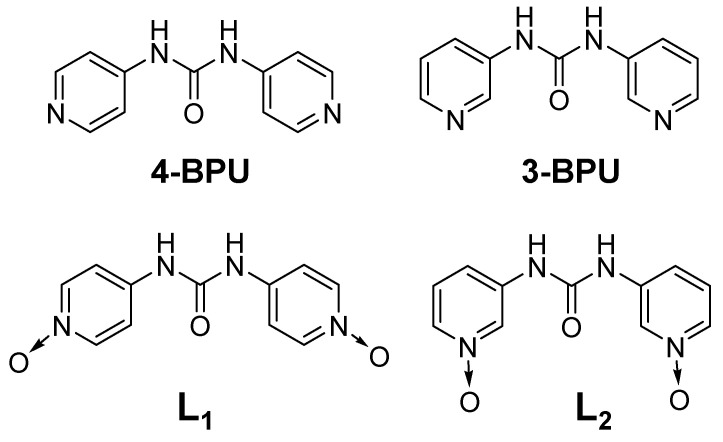
Parent bis(pyridyl) urea and the corresponding *N*–oxide compounds obtained by modifying the pyridyl groups.

The reaction of 4–aminopyridine/3–aminopyridine with triphosgene in the presence of triethylamine in anhydrous dichloromethane resulted in *N*,*N*’–bis(4–pyridyl) urea (**4–BPU**)/*N*,*N*’–bis(3–pyridyl) urea (**3–BPU**) [32]. The **3–BPU** did not form hydrogels in pure water and the majority of aqueous solutions whereas **4–BPU** formed gels in water/aqueous solutions and the structural details of **4–BPU** interacting with the gelling solvents has been reported [32]. The structural modifications of the bis(pyridyl) urea compounds were achieved by oxidizing the pyridyl groups of **4–BPU** and **3–BPU** to pyridyl *N*–oxide moieties using 3–chloroperoxybenzic acid, resulting in di–*N*–oxide urea 4,4’–(carbonylbis(azanediyl))bis(pyridine 1–oxide) (**L_1_**) and 3,3’–(carbonylbis(azanediyl))bis(pyridine 1–oxide) (**L_2_**), respectively (Scheme 1).

### 2.2. Gelation Experiments

The gelation property of **4–BPU** was previously reported [32] and we have repeated the gelation studies at 1.0 wt% (wt% refers to wt/v% in all cases) in water and various solvent/water mixtures (Table S1). Since **3–BPU** was reported to be non-hydrogelator, we have analyzed the gelation properties in mixed aqueous solutions (Table S1) and found that **3–BPU** formed a gel in a mixture of water and ethylene glycol (EG) or 1,2–dimethoxyethane (DME) at 3.0 wt%. The gelation experiments were repeated by varying the ratio of EG or DME in water, which indicated that a 3:7 (*v*/*v*) ratio of EG/water or DME/water was the optimum concentration to obtain the gel. Thus, we have chosen water and a mixture of EG/water (3:7, *v*/*v*) as the standard solvents to compare the gelation of the parent and modified gelators.

**Figure 1:**
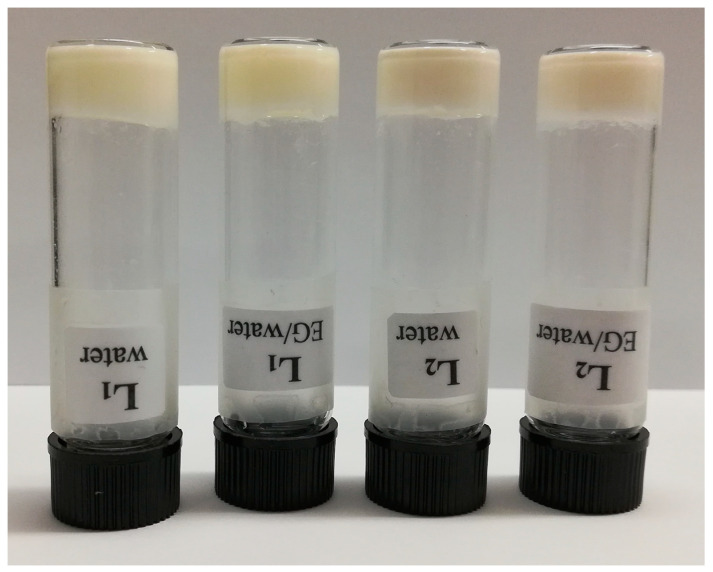
**L_1_** and **L_2_** gels obtained from water and EG/water (3:7 *v*/*v*) mixture.

The compound **L_1_** was found to be sparingly soluble in common organic solvents but **L_2_** was partially soluble at room temperature. The experiments to test gel formation in water resulted in hydrogels of both **L_1_** and **L_2_** at 1.0 wt% (Figure 1). Due to the insolubility of **L_1_** at a higher concentration (>2.0 wt%) in water, we checked the gelation ability of **L_1_** in aqueous solutions of highly polar solvents. The gelation was tested in nine different solvent/water mixtures (1:1, *v*/*v*) such as DMF, DMA, DMSO, EG, DME, MeOH, EtOH, MeCN, or THF at 1.0 wt% and the experiments were performed by dissolving **L_1_** in the mixed solvent by heating, sonicated for a few seconds and left undisturbed. The gelation experiments performed in 1:1 (*v*/*v*) mixture of DMSO/water, DMF/water, DMA/water, EG/water, and DME/water resulted in gel formation within an hour, but gelation was not observed in other solvent mixtures (Table 1). The gelation properties were studied by varying the concentration of EG in EG/water mixtures and the results indicated that decreasing the concentration of the water (<50.0%) prevented gel formation.

The minimum gel concentration (MGC) of **L_1_** was determined by varying the concentration of gelator and the MGC was found to be 0.7 wt% in both water (Table S2) and EG/water mixture (3:7, *v*/*v*). The MGC of the parent **4–BPU** gelator was found to be 0.8 wt% in water and 0.7 wt% in EG/water mixture (3:7, *v*/*v*). These results clearly indicated that **4–BPU** and the modified compound **L_1_** can form the entangled network, which showed the important role of urea moieties in gel formation. We have also tested the gelation properties of **L_2_**, which was synthesized by modifying **3–BPU**. The solubility of **L_2_** was higher compared to **L_1_** in water or an aqueous solution of organic solvents. The ability of **L_2_** to form hydrogel was tested by dissolving 10.0 mg of **L_2_** in 1.0 mL solvent by heating and sonicating, and the solution was left undisturbed. Interestingly, the hydrogel was obtained within an hour (Table 1) and the MGC of **L_2_** in water was found to be 0.8 wt% (Table S2). Thus, replacing the pyridyl groups with *N*–oxide groups induced hydrogel formation in **L_2_**, which was not observed in parent **3–BPU**. The gelation experiments performed in (1:1, *v*/*v*) aqueous solutions of highly polar solvents (EG, DME, DMF, DMA, DMSO, MeOH, EtOH, MeCN and THF) at 1.0 wt% resulted in gelation of **L_2_** in all cases. The MGC of **L_2_** in the EG/water mixture (3:7, *v*/*v*) was found to be 1.1 wt%. We have also performed gelation experiments by varying the concentration of ethylene glycol and the results showed that increasing the concentration of EG in the EG/water mixture increased the MGC of **L_2_** gels, indicating that EG acts as the solubilizing agent.

The thermal stabilities of the gel networks were evaluated by gel-to-solution transition temperature (*T_gel_*) experiments. The thermal stabilities of **4–BPU**, **3–BPU**, **L_1_**, and **L_2_** were measured in water and various EG/water ratios, and the results are summarized in Table 2. The *T_gel_* comparison of **4–BPU** and **L_1_** revealed that both the networks displayed similar thermal stabilities in pure water. However, increasing the EG concentration resulted in reduced thermal stability for all the gelators, which may be attributed to the higher solubility of the gelators in EG. The analysis of the thermal stabilities at various solvent compositions showed that **L_1_** displayed enhanced thermal stabilities compared to **4–BPU** (Table 2). The *T_gel_* comparison of **3–BPU** and **L_2_** clearly indicate that the thermal stability of the **L_2_** network is much higher compared to **3–BPU**. Furthermore, **L_2_** is capable of forming gel in a wide range of solvent compositions. Thus, modifying the pyridyl groups of bis(pyridyl) urea compounds have a prominent effect on the thermal stability of the gel network.

### 2.3. Rheology

The structural characteristics of the bis(pyridyl) and bis(pyridyl–*N*–oxide) urea gels were analyzed by rheology, which enabled us to compare the relative stiffness or elasticity of the gel networks. The **4–BPU**, **L_1_** and **L_2_** gels were prepared at 1.0 wt% in water, and the linear viscoelastic region (LVR) was determined by oscillatory strain−sweep experiments, where the elastic modulus (G’) was constant irrespective of the shear stress. The experiments were performed within the LVR to ensure that the gel networks underwent reversible deformation, which would enable us to estimate the actual characteristics of the materials. The **4–BPU** gel displayed quite narrow LVR, and the G’ decreased on increasing the shear strain above 0.05% (Figure S1).

**Figure 2:**
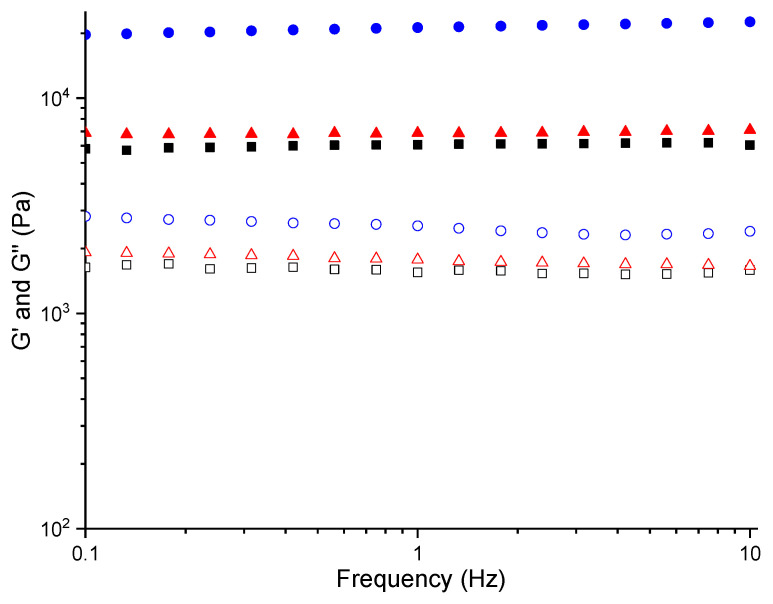
Frequency sweep experiments performed on **4–BPU**, **L_1_** and **L_2_** gels at 1.0 wt% in water at 25.0 °C at a constant strain of 0.05%. Color codes: G’, **4–BPU** (■), G’’, **4–BPU** (□), G’, **L_1_** (▲), G’’, **L_1_** (Δ), G’, **L_2_** (●), G’’, **L_2_** (**○**).

In contrast, the **L_1_** and **L_2_** gels were more rigid, and the LVR of these gels were stretched to 0.1% (Figure S1). The cross-over point, where the solid-like property of the gel transformed to a liquid-like property was also higher for the **L_1_** and **L_2_** gels. These indicate that the *N*–oxide gels are capable of resisting higher applied forces without irreversible deformation compared to the **4–BPU** gel. The frequency sweep experiments performed on the gels at 1.0 wt% in water revealed that both of the storage and loss moduli (G’ and G’’) were independent of frequency, supporting the gel behavior (Figure 2). The **4–BPU** and **L_1_** gels displayed quite similar G’, but the G’ of **L_2_** gel was significantly higher, indicating that the **L_2_** gel possessed a stronger network with higher mechanical stability. The relative gel strength of **3–BPU** and **L_2_** was also compared by performing rheology on the gels prepared at 2.5 wt% in EG/water (3:7 *v*/*v*). The strain sweep (Figure S1) and frequency sweep (Figure S2) experiments performed on both gels revealed that **L_2_** displayed about 1000 times higher G’ compared to the parent **3–BPU**. The G’ and G’’ of **3–BPU** did not show a constant value at low-frequency range, indicating that the **3–BPU** gel network was soft resulting in a weak solid-like network. This is in excellent agreement with the overall gelation properties of the compounds, which suggested that **4–BPU**, **L_1_** and **L_2_** were efficient hydrogelators but that **3–BPU** was a weak gelator.

### 2.4. Gel Morphology

The morphologies of **4–BPU**, **3–BPU**, **L_1_**, and **L_2_** were studied by scanning electron microscopy (SEM). The **4–BPU** (1.0 wt%), **L_1_** (1.0 wt%), **3–BPU** (2.5 wt%) and **L_2_** (2.5 wt%) gels were prepared from EG/water (3:7, *v*/*v*). We have also performed the SEM analysis of **L_1_** and **L_2_** at 1.0 wt% in water and DMSO/water (1:1, *v*/*v*). The gels were filtered to remove the solvent and dried for two days to prepare the xerogel and the SEM images of the dried gels indicated the presence of various morphologies (needle, fibrous, tape-like and plate).

**Figure 3:**
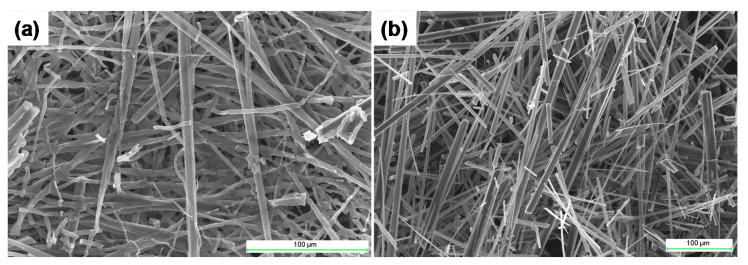
SEM images of (**a**) **4–BPU** and (**b**) **L_1_** xerogels obtained from EG/water (3:7, *v*/*v*) at 1.0 wt%.

**Figure 4:**
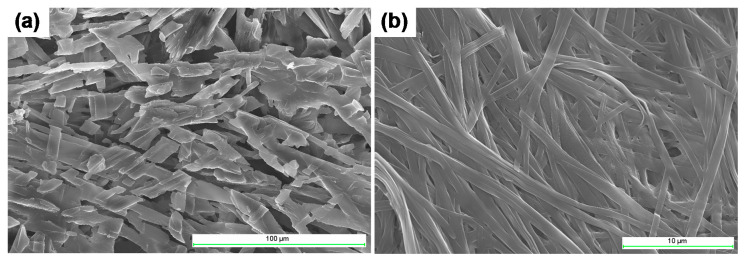
SEM images of (**a**) **3–BPU** and (**b**) **L_2_** xerogels obtained from EG/water (3:7, *v*/*v*) at 2.5 wt%.

The **4–BPU** xerogels obtained from EG/water (3:7, *v*/*v*) displayed a typical fibrous network with thickness ranging from 2.0 to 8.0 µm (Figure 3a). The morphology of **L_1_** gels from both EG/water mixture (Figure 3b) and pure water (Figure S3) was slightly different from the parent **4–BPU** gelator. The xerogels of **L_1_** displayed needle-like fibers with thickness ranging from 4.0 to 12.0 µm whereas a tape-like fibrous network was observed in **4–BPU**. The xerogel of **L_1_** (1.0 wt%) obtained from DMSO/water mixture (1:1, *v*/*v*) showed a fibrous tape-like morphology with thickness varying from 0.1 to 1.6 µm (Figure S4). The xerogels of **3–BPU** from EG/water (3:7, *v*/*v*) displayed a fibrous brick-like morphology (Figure 4a) varying from 5.0 to 20.0 µm but a different morphology was observed for **L_2_** xerogels in both EG/water (3:7 *v*/*v*) (Figure 4b) and pure water (Figure S5).

The thickness of the fibers observed for **L_2_** xerogels from EG/water, 3:7 *v*/*v* and pure water varied from 0.8 to 1.2 µm and 0.2 to 0.8 µm, respectively. The SEM images of **L_2_** (1.0 wt%) xerogel obtained from 1:1 DMSO/water mixture (*v*/*v*) showed morphologies similar to EG/water gels with varying thickness (0.1 to 1.0 µm, Figure S6). These results clearly indicate that modifying the pyridyl group to *N*–oxide groups induced a prominent change in the morphology of the gel fibers. Thus, the mode of intermolecular nonbonding interactions plays an important role in altering the morphology of gel fibers, which prompted us to correlate the solid-state structure of the modified compounds with the parent gelator structure using X-ray diffraction.

### 2.5. Single Crystal X-ray Diffraction

**Figure 5:**
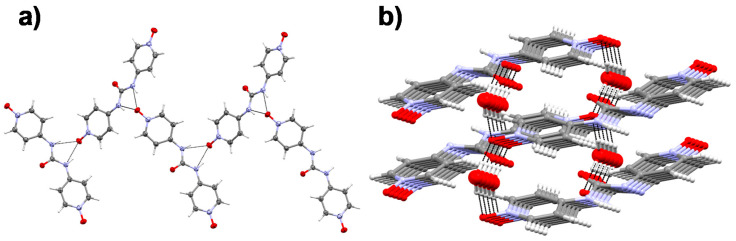
(**a**) Hydrogen bonded 1-D chain observed in **L_1_** and (**b**) 1-D chains displaying bifurcated hydrogen bonding with adjacent chains and solvent water molecules via O—H∙∙∙O interactions.

The solid-state structures of the parent gelator **4–BPU [32]**, the isomer **3–BPU** [32], and the *N*–oxide **L_2_.H_2_O [42]** were compared to the modified **4–BPU** gelator (**L_1_**). We were successful in isolating the crystals of the gelators in various solvated forms such as **L_1_.H_2_O**, **3–BPU.2EG**, and **L_2_.EG**. Single crystals of **L_1_** were obtained from an aqueous solution of **L_1_** below MGC over a period two days. The crystal data and the hydrogen-bonding parameters are summarized in Tables S3 and S4, respectively. Single crystal X-ray diffraction revealed that **L_1_** crystallized in a monoclinic space group *P2_1_*/*c* with one solvent water molecule (**L_1_.H_2_O**). The molecular plane consisted of one of the pyridyl *N*–oxide moieties and the urea moiety, but the second pyridyl moiety was slightly twisted from the molecular plane (15.6°). The hydrogen bonding modes of the two pyridyl *N*–oxide moieties were different and the twisted pyridyl *N*–oxide moiety displayed N—H∙∙∙O interaction (2.7990(16) and 2.7851(16) Å) with the urea moiety, which was similar to complementary urea interaction. This interaction resulted in a one-dimensional chain with **L_1_** orienting orthogonal to each other displaying urea *α*−tape-like architecture (Figure 5a). The 1-D chains displayed bifurcated hydrogen bonding with adjacent chains via O—H∙∙∙O interactions (2.7466(19) and 2.7612(19) Å) involving the planar pyridyl *N*–oxide moiety and the solvent water molecule (Figure 5b).

The analysis of the solid-state structure of **L_1_** and the parent gelator (**4BPU**) revealed the absence of complementary N—H∙∙∙O=C hydrogen bonding motifs in **4BPU** [32] because the pyridyl groups and the urea moieties were hydrogen bonded with the solvent molecules. The structural analysis of the crystals of **L_2_** revealed the formation of the solvated form of **L_2_** (**L_2_**.**H_2_O**) and the unit cell parameters matched with the reported structure [42]. The hydrogen bonding pattern of the pyridyl *N*–oxide and urea moieties of **L_2_** was similar to **L_1_** and the one-dimensional hydrogen-bonded chain formed was stabilized by the complementary hydrogen bonding [42]. The structure was compared with the parent compound **3–BPU [32]**, which existed in two forms (**3–BPU** and **3–BPU.H_2_O**). The pyridyl groups of **3–BPU** were involved in N—H∙∙∙N interactions and these groups were hydrogen bonded to the solvent water molecules in **3–BPU.H_2_O [32]**. Thus, replacing the nonbonding interactions resulted in a one-dimensional hydrogen bonded chain in **L_1_** and **L_2_** stabilized by complementary urea-like hydrogen bonding (N—H∙∙∙O). Moreover, the alteration of the pyridyl group to the pyridyl *N*–oxide moiety induced gelation resulting in **L_2_** hydrogels but the parent **3–BPU** was a nongelator in water.

**Figure 6:**
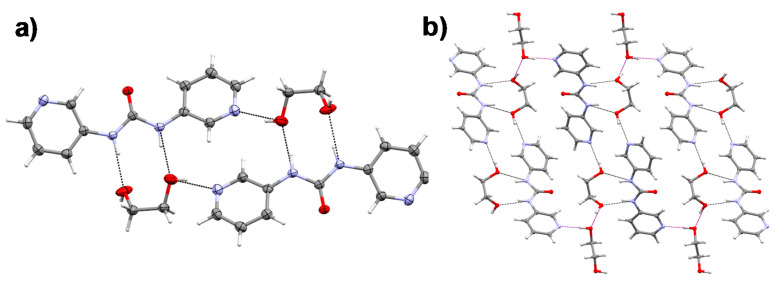
(**a**) Hydrogen-bonded macrocycle obtained by the interaction of solvent ethylene glycol and **BPU** molecules in **BPU.2EG**, (**b**) macrocycle interacting with adjacent macrocycles via O—H∙∙∙O interactions.

**Figure 7:**
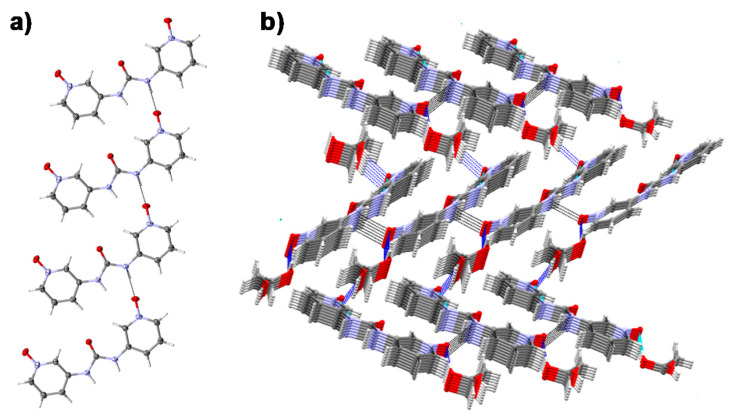
(**a**) Hydrogen bonded chains in **L_2_**.**EG** stabilized by N—H∙∙∙O interactions between urea and pyridyl *N*–oxide moieties, (**b**) one-dimensional chains interacting with adjacent chains via N—H∙∙∙O interaction involving pyridyl *N*–oxide groups and the other N—H groups of the urea moiety.

The gelation ability of **3–BPU**, **L_1_**, and **L_2_** in EG/water prompted us to analyze the crystal structure of these compounds obtained from ethylene glycol. The structural analysis of **3–BPU** obtained from ethylene glycol revealed that **3–BPU** crystallized in monoclinic space group *P2_1_*/*c* with two EG molecules (**3–BPU.2EG)**. The pyridyl nitrogen atoms were oriented trans to each other resulting in an anti-conformation compared to the reported syn-conformation of **3–BPU** and **3–BPU.H_2_O** structures [32]. The urea moiety and the pyridyl nitrogen atom of **BPU.2EG** were hydrogen bonded to one of the EG molecules via N—H∙∙∙O (2.9122(14) and 2.7496(13) Å) and O—H∙∙∙N (2.7932(15) Å) interactions resulting in a hydrogen bonded macrocycle (Figure 6a). This macrocycle interacts with adjacent macrocycles via O—H∙∙∙O interactions (2.7026(15) Å) between the other pyridyl moieties and the second EG molecule to form a two-dimensional hydrogen bonded corrugated sheet-like architecture (Figure 6b). The modified compound **L_2_** crystallized in an orthorhombic *C222_1_* space group with a formula of **L_2_**.**EG** and a syn-conformation of pyridyl *N*–oxide was observed. One of the N—H groups of the urea moiety interacted with the pyridyl *N*–oxide group via a complementary urea-like hydrogen bonding (N—H∙∙∙O = 2.690(2) Å) interaction similar to **L_2_**.**H_2_O** resulting in a one-dimensional chain (Figure 7a), which interacted with adjacent chains via N—H∙∙∙O interaction (2.690(2) Å) between the pyridyl *N*–oxide groups and the other NH groups of the urea moiety. The pyridyl *N*–oxide moiety also interacted with the EG molecule via O—H∙∙∙O (2.741(2) Å) hydrogen bonding interaction (Figure 7b) resulting in a three-dimensional hydrogen bonded network.

### 2.6. X-ray Powder Diffraction (XRPD)

The molecular aggregation in the solid-state and gel/dried gel state was compared by the XRPD analysis of the bulk material and the xerogel. We have been successful in identifying the role of various nonbonding interactions in gel formation by comparing the molecular assembly in crystalline and gel/dried gel states [30,43,44,45]. Although, the xerogel does not represent the exact structure of the gel state since the drying process can lead to a morphological change or phase transition [46], this approach is still recognized as the most useful strategy to unravel the self-assembly of a gelator [3,7,29,30,43,44,45]. The self-assembly of **L_1_** and **L_2_** in the gel state was correlated with respective crystal structures by comparing the powder X-ray pattern of the bulk crystals and dried gels with the crystal structure. The XRPD pattern for the bulk material and xerogel pattern matched with the simulated graph obtained from the single-crystal structure of **L_1_** (Figure S7). This indicates that the single crystal structure of **L_1_** truly represents the packing in bulk crystal and the hierarchical xerogel network. We have performed the XRPD of xerogels obtained from water and EG/water mixture (3:7, *v*/*v*), and the pattern matched each other but the xerogel pattern obtained from water was less crystalline indicating that EG induced a crystalline nature in gel fibers (Figure S7).

**Figure 8:**
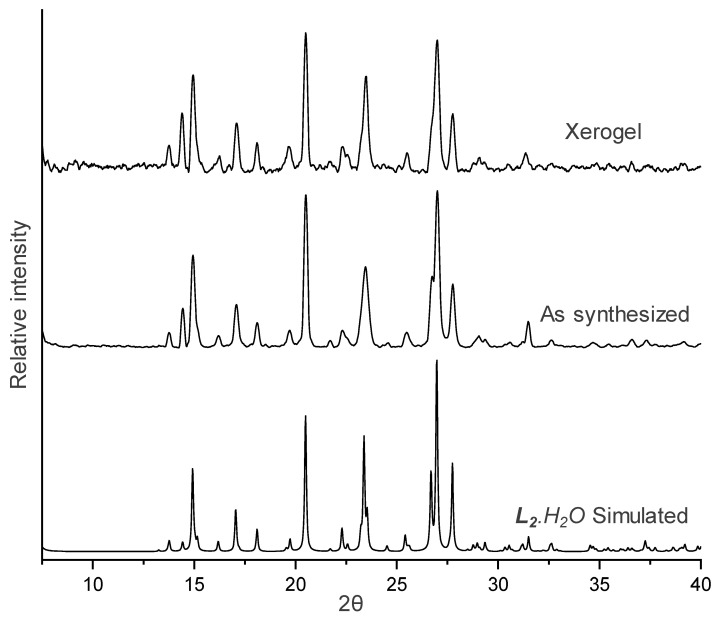
Comparison of the XRPD pattern of simulated, as synthesized and the xerogel from EG/water (3:7 *v*/*v*) at 1.2 wt% of **L_2_**.

Similar results were observed for **L_2_**, the XRPD of the **L_2_** xerogels obtained from EG/water mixture (3:7, *v*/*v*) and bulk solid were compared with the simulated pattern of **L_2_.H_2_O** and the powder pattern of both bulk crystals and xerogel matched with the simulated pattern of **L_2_.H_2_O** (Figure 8). Interestingly, the bulk crystals of **L_2_.EG** from EG did not match the **L_2_.EG** simulated pattern (Figure S8), but matched perfectly with **L_2_.H_2_O** pattern, presumably due to the loss of hydrogen bonded EG molecules during the drying process. We observed similar trends for **3–BPU**, where XRPD pattern of **3–BPU.EG** bulk crystals and xerogels obtained from EG/water mixture (3:7, *v*/*v*) showed a perfect match with the simulated **3–BPU** pattern (Figure S9) but these patterns were different from the simulated **3–BPU.EG** pattern. This confirms the loss of EG molecules in the crystal during the drying process. The xerogel patterns obtained for **L_1_** and **L_2_** from water and EG/water mixture (3:7, *v*/*v*) indicates that the one-dimensional urea α−tape-like architecture was preserved in the xerogel network.

### 2.7. Stimuli-Responsive Property

**Figure 9:**
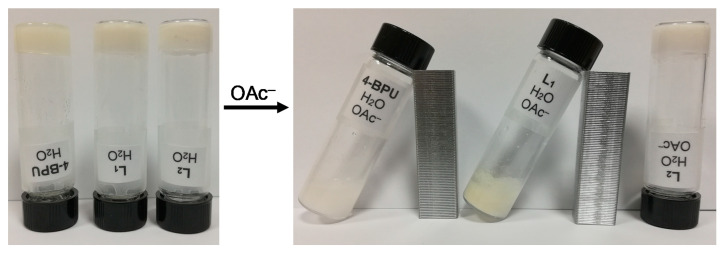
The effect of four equivalents of acetate anion on **4–BPU**, **L_1_** and **L_2_** gels.

The amide/urea functionalized LMWGs can be considered as classical stimuli-responsive soft materials due to their response to an external stimulus such as light, redox, pH, and salts/ions [29,37,43,47]. We have shown that the anion responsive properties of supramolecular gels can be used to detect cyanide anions in water by monitoring the gel to sol transition [44]. The stimuli-responsive properties of the gels were studied by treating the hydrogels of **4–BPU**, **L_1_**, and **L_2_** at 1.0 wt% with different anions such as halides, acetate, and cyanide (sodium or potassium salt), and the relative gel strength of the mixture was compared with the pure gels. The **4–BPU** gel was stable with the addition of three equivalents of either fluoride, chloride, bromide, iodide, acetate, and cyanide salts (Figure S10). However, the network was collapsed by the addition of four equivalents of iodide, acetate, and cyanide ions (Figure 9). The gel was also disrupted by the addition of excess bromide (five equivalents), fluoride (six equivalents), and chloride ions (seven equivalents). This indicates that larger anions were more effective in breaking the gel network, presumably due to the enhanced interactions with the N—H∙∙∙N hydrogen bonding synthons. The higher efficiency of fluoride in disrupting the gel network compared to the chloride ion may be attributed to the strong hydrogen bonding capability of the fluoride ions. The experiments performed with the modified gelator showed that **L_1_** was more sensitive to the anions (Figure S11), and the gel was collapsed by the addition of two equivalents of iodide and cyanide ions. The gel network was also collapsed in the presence of three equivalents of bromide and four equivalents of acetate ions (Figure 9). These results clearly indicate that **L_1_** gel is more responsive compared to the parent **4–BPU** gel.

**Figure 10:**
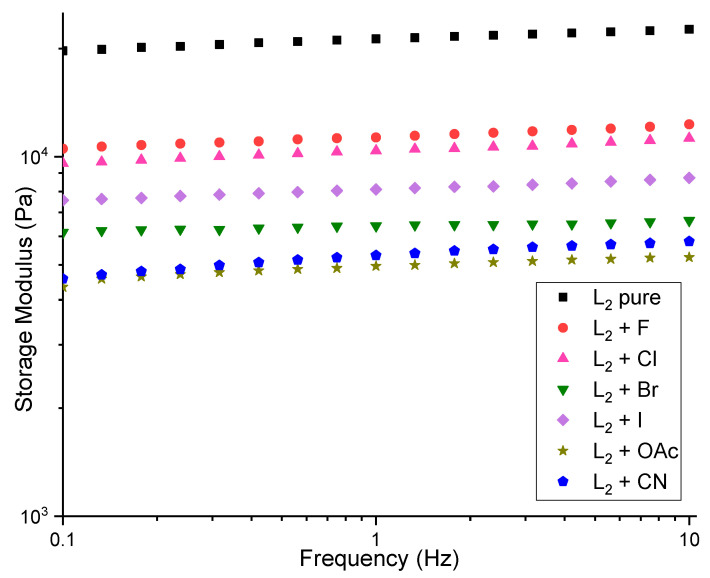
Frequency sweep experiments performed at 25.0 °C at a constant strain of 0.02% on **L_2_** hydrogel at 1.0 wt%, in the presence of six equivalents of anions.

Interestingly, the **L_2_** hydrogel displayed much higher stability towards anions compared to **4–BPU** and **L_1_** gels, and the gel network was stable even after the addition of six equivalents of anions in all cases. This was due to the rigid network of **L_2_** compared to **4–BPU** and **L_1_** gels, thus **L_2_** exhibited more resistance against the anions. The effect of the anions on the gel network was further analyzed by rheology. The addition of three equivalents of anions to the **L_2_** gel resulted in the reduction of the storage modulus, however, no significant difference in the G’ value was observed with various anions (Figure S12). The G’ of the gels decreased by increasing the concentration of the anions (six equivalents) for bromide, iodide, acetate, and cyanide ions (Figure 10). The larger ions such as acetate and cyanide showed the lowest G’ values for **L_2_**, which corroborates the experiments with **4–BPU** and **L_1_** gels. The analysis of the anion sensing experiments suggests that the modification of the functional groups can potentially generate gels with higher sensitivity and resistance.

### 2.8. Computational Studies

To rationalize these observations, we have calculated the strengths of different hydrogen-bonding interactions using quantum chemical calculations via a state-of-the-art density functional theory (DFT)-based protocol (⍵B97M–D3BJ/def2–TZVP). Interactions of gelators/nongelators with themselves as well as with single solvent molecules EG and water molecules were calculated. These calculations performed in the gas phase allowed us to compare the hydrogen-bonding interaction strength of different functional groups, which were not intended, however, to mimic the complex solution/gel environment. Figures S13–S16 (see Supplementary Materials) show the optimized geometries as well as interaction energies. Calculations of the nongelator **3BPU** revealed that the strongest **3BPU** dimer interaction was −17.5 kcal/mol (ΔE) and involved N—H···O hydrogen-bonding. This was almost equal to the **3BPU**–EG interaction of –17.8 kcal/mol. The **4–BPU** dimer can hydrogen-bond via either N—H···O or N—H···N. The stronger N—H···O interaction within the **4–BPU** dimer (−15.4 kcal/mol) was, however, calculated to be weaker than the N—H···O interaction between **4BPU** and EG (–16.6 kcal/mol). The hydrogen-bonding interactions with water and **3–BPU**/**4–BPU** were generally calculated to be weaker than **3–BPU**/**4–BPU** dimer interactions but interactions with EG were predicted to be strongest overall.

Calculations of **L_1_** and **L_2_** dimers revealed a rather different picture. The **L_1_**–**L_1_** dimer interaction via N—H···O hydrogen bond was rather a stronger interaction (−19.9 kcal/mol) that can be compared to the relatively weak H_2_O–**L_2_** O—H···O—N interaction (−10.4 kcal/mol) and the EG–**L_1_** N—H···O interaction (−16.6 kcal/mol). For the **L_2_**–**L_2_** dimer we found two different conformations. For example, one with an approximately parallel alignment of the **L_2_** units and another with more perpendicular alignment. The parallel conformation had the interaction energy of −19.6 kcal/mol while the perpendicular conformation had the interaction energy of −24.3 kcal/mol. Both of these conformations were found to be stronger than **L_2_**–EG interactions (−15.1 kcal/mol) and an **L_2_**.H_2_O interaction (−8.8 kcal/mol). These simple hydrogen-bonding calculations in the gas phase do not explain why **4–BPU** was a gelator and **3–BPU** a non-hydrogelator. More complex calculations that take the solution/gel environment into account are likely required to understand the nature of the gelation process. The increased dimer hydrogen-bonding interaction strength in going from **3–BPU** to **L_2_** via pyridyl nitrogen to *N*–oxide substitution is, however, a plausible model for why **L_2_** formed the better gels than **3–BPU**.

## 3. Conclusions

The structural modification of the gelator/nongelator was used as a strategy to alter the nonbonding interactions responsible for gel formation to induce/enhance the gelation properties of LMWGs. This was achieved by converting the pyridyl moieties of a bis(pyridyl) urea-based hydrogelator (**4–BPU**) and a non-hydrogelator (**3–BPU**) to pyridyl *N*–oxides compounds (**L_1_** and **L_2_**, respectively). The gelation properties of the modified compounds and the parent compounds were analyzed by standard gelation techniques and improved thermal stability was observed for **L_1_** compared to the parent **4–BPU**. The hydrogel formation of **L_2_** clearly indicates that the structural modification of **3–BPU** induced gelation. The modified gelator **L_2_** turned out to be an excellent gelator in water and mixed aqueous solvents. The modification of the functional groups had a prominent effect on the mechanical and thermal stability of the gel network and the modified gelators showed higher sol–gel transition temperatures (*T_gel_*). The morphologies of the xerogels were analyzed by comparing the SEM images, which showed that brick-like morphology of **3–BPU** was replaced by an efficient fibrous network in **L_2_** in water, which corroborates well with the gelation results. The comparison of the solid-state structures revealed that a one-dimensional chain was formed via N—H∙∙∙O interaction similar to complementary urea interaction, which was crucial for the formation of the gel network. The enhanced gelation properties resulting from the alteration of N—H∙∙∙N interaction to N—H∙∙∙O interaction were also supported by the rheological measurements, which established the stiffer network of the modified gelators. The stimuli-responsive property was studied by adding various anions to the hydrogels, which revealed that **L_1_** gel was a better anion sensor whereas **L_2_** gel displayed significant resistance towards anions. Density functional theory calculations confirmed the increased hydrogen bonding strength of N—H∙∙∙O vs. N—H∙∙∙N interactions. This implies that the spatial arrangement of the functional groups and the nature of intermolecular nonbonding interactions of the gelators are crucial for the three-dimensional arrangement of the gel network.

## 4. Materials and Methods

All starting materials and solvents were purchased from commercial sources and used as supplied. Deionized water was used for all the experiments and anhydrous dichloromethane was obtained by distilling the solvent over CaH_2_. The **3–BPU** and **4–BPU** were synthesized following the reported procedure [32] and the analytical data matched with the reported compounds. ^1^H and ^13^C NMR spectra were recorded on a Bruker Advance 400 spectrometer (^1^H–NMR: 400 MHz, ^13^C–NMR: 100 MHz) and SEM was performed on a Leo Supra 25 microscope.

### 4.1. Synthesis of the Ligand

#### 4.1.1. 4,4’–(carbonylbis(azanediyl))bis(pyridine 1–oxide) (**L**_1_)

To a solution of **4–BPU** (1.1 g, 5.1 mmol) in DMF (30 mL), 3–chloroperoxybenzic acid (3.4 g, 14.8 mmol) was added in portions over a period of 15 min at room temperature and the mixture was stirred overnight. The white precipitate obtained was filtered and stirred overnight in 0.05 *N* HCl. The mixture was filtered, washed with water and dried to obtain **L_1_** as a white powder. The product was recrystallized from water. Yield = 0.62 g (2.5 mmol, 49.4%). ^1^H–NMR (400 MHz, DMSO–*d_6_*): δ = 9.45 (2H, s), 8.10 (4H, d, J = 8.0), 7.49 (4H, d, J = 8.0). ^13^C–NMR (100 MHz, DMSO–*d_6_*): δ = 151.47, 138.76, 136.66, 115.25. MS (ESI) m/z for C_11_H_10_N_4_O_3_Na^+^: expected 269.0651, found 269.0663.

#### 4.1.2. 3,3’–(carbonylbis(azanediyl))bis(pyridine 1–oxide) (**L**_2_)

**L_2_** was synthesized following a similar procedure as above by reacting **3–BPU** (1.1 g, 5.1 mmol) and 3–chloroperoxybenzic acid (3.4 g, 14.8 mmol) in 30 mL DMF. The product was recrystallized from methanol and all gelation experiments were performed with the recrystallized product. Yield = 0.72 g (2.9 mmol, 56.5%). ^1^H–NMR (400 MHz, DMSO–*d_6_*): δ = 9.31 (2H, s), 8.53 (2H, d, J = 4.0), 7.92 (2H, m), 7.33–7.34 (4H, m). ^13^C–NMR (100 MHz, DMSO–*d_6_*): δ = 151.83, 138.49, 132.88, 129.52, 126.06, 115.45. MS (ESI) m/z for C_11_H_10_N_4_O_3_Na^+^: expected 269.0651, found 269.0659.

### 4.2. Gelation Studies

#### 4.2.1. Gelation Test

The gelation properties of **L_1_** and **L_2_** were tested in various solvents by adding 1.0 mL of the solvent to 10.0 mg of *N*–oxide compounds (1.0 wt%) in a standard 7.0 mL vial and the mixture was heated to obtain a clear solution. The solution was sonicated, left undisturbed at room temperature and gelation was confirmed by inversion test. The gelation experiments in a mixed solvent system (1:1, *v*/*v*) were performed by dissolving the gelator in 500 µL of the appropriate solvent and distilled water (500 µL) was added as cosolvent. The mixture was heated to obtain a clear solution, cooled to room temperature and left undisturbed for gel formation.

#### 4.2.2. Minimum Gel Concentration

The MGC experiment was performed by weighing various amounts of gelator in a standard 7.0 mL vial, followed by adding 1.0 mL of solvent (or solvent mixture). The ligand was dissolved by heating the mixture and the solution was sonicated and left at room temperature for gelation. The minimum concentration at which the gel was obtained after 24 h was recorded as MGC.

#### 4.2.3. T_gel_ Experiments

The gel to solution transition temperature (*T_gel_*) was evaluated in different solvent systems using ball-drop method [7,30,44,45]. The required amount of the gelator was dissolved in the solvent or solvent mixtures by heating the mixture in a sealed vial to obtain a clear solution. The solution was cooled to room temperature and after 24 h, a spherical glass ball (52.0 mg) was placed on top of the gel. The vial was placed into an oil bath equipped with a magnetic stirrer and a thermometer. The oil bath was gradually heated and the temperature at which the glass ball touched the bottom of the vial was recorded as *T_gel_*.

### 4.3. Rheology

Rheological measurements were performed in an Anton Paar MCR 302 rheometer using a 25.0 mm stainless steel parallel plate geometry configuration. The **4–BPU**, **L_1_**, and **L_2_** gels were prepared by dissolving 10.0 mg of the compound in 1.0 mL of water and experiments were performed by scooping a ~1.0 mL portion of gel onto the plate. The **3–BPU** and **L_2_** gels were also prepared at 2.5 wt% in EG/water (3:7 *v*/*v*). Viscoelastic properties were evaluated by oscillatory measurements at a constant temperature of 25.0 °C. Amplitude sweeps were performed with constant frequency (f) of 1.0 Hz and log ramp strain (γ) = 0.01–100% and frequency sweeps were carried out between 0.1 and 10.0 Hz within the linear viscoelasticity domain (0.05% strain). The anion sensing experiments were performed by dissolving the mixture of gelator and anions (sodium or potassium salts) in water by heating and sonicating. The mixture was cooled to form gels, the gels were scooped after 24 h, and rheology was performed similarly.

### 4.4. Scanning Electron Microscopy

The **4–BPU** and **3–BPU** gels were prepared from EG/water (3:7, *v*/*v*) at 1.0 wt% and 2.5 wt% respectively. The gels of **L_1_** (1.0 wt%) and **L_2_** (2.5 wt%) were prepared in water and EG/water (3:7, *v*/*v*), and 1.0 wt% gels were prepared for both **L_1_** and **L_2_** in DMSO/water (1:1, *v*/*v*). The resulting gels were filtered after 24 h and dried under a fume hood. A small portion of the dried gels was placed on a pin mount with a carbon tab on top and coated with gold for 2 min. SEM images of the dried gels were analyzed using Leo Supra 25 microscope.

### 4.5. Single Crystal X-ray Diffraction

X-ray quality single crystals of **3–BPU**, **L_1_** and **L_2_** were obtained by the slow evaporation of the compounds from corresponding solvents. Needle-shaped crystals of **L_1_.H_2_O** were obtained by the slow evaporation of an aqueous solution (2.0 mL) of **L_1_** (10.0 mg) in an open vial. The crystallization experiment of **L_2_** in ethylene glycol was performed by dissolving 20.0 mg of **L_2_** in 1.0 mL of EG and left for slow evaporation under the fume hood. Concomitant crystals of block-shaped **L_2_.H_2_O** and plate shaped **L_2_.EG** were obtained in three days. The crystallization experiment performed with **3–BPU** in EG resulted in long needle-shaped crystals in two days, which were obtained by the slow evaporation of 40.0 mg of **3–BPU** in 1.0 mL of ethylene glycol. The crystals were isolated from the solvent and immersed in cryogenic oil before mounting. The crystals were mounted on a Bruker D8 VENTURE (Photon100 CMOS detector) diffractometer equipped with a Cryostream open-flow nitrogen cryostat and the data collection were performed using CuK_α_ radiation (λ = 1.54178 Å) at 150.0(2) K. The unit cell determination, data collection, data reduction, structure solution/refinement, and empirical absorption correction (SADABS) were carried out using Apex III. The structure was solved by a direct method and refined by the full-matrix least-squares on F^2^ for all data using SHELXTL version 2017/1 [48]. All non-disordered nonhydrogen atoms were refined anisotropically, and the hydrogen atoms were placed in the calculated positions and refined using a riding model except for **L_1_.H_2_O** where the hydrogen atoms of the water molecules were located on the Fourier map and refined. The free variables of disordered carbon atoms in the EG molecule in **3–BPU.2EG** were refined by the FVAR instruction. The crystallographic data for this paper were deposited at Cambridge Crystallographic Data Centre and the CCDC numbers are 1965865–1965867. These data can be obtained free of charge via www.ccdc.cam.ac.uk/data_request/cif, or by emailing data_request@ccdc.cam.ac.uk.

### 4.6. X-ray Powder Diffraction

The bulk crystals of **L_1_** were obtained by the slow evaporation of the solution of **L_1_** (10.0 mg in 2.0 mL water). The gelator **L_2_** (20.0 mg) was dissolved in 1.0 mL of EG and left under a fume hood for crystallization resulting in bulk crystals of **L_2_**. The crystals of 3–BPU were obtained from ethylene glycol solution (40.0 mg in 1.0 mL). The crystals were filtered, dried in the air, and ground to a fine powder. The xerogels of **3–BPU**, **4–BPU**, **L_1_**, and **L_2_** were prepared by filtering the corresponding gels in water or in 3:7 EG/water (*v*/*v*) mixture at a concentration closer to MGC, followed by drying the residue in a fume hood. XRPD was carried out on the bulk crystals and xerogels using a Bruker D8 Focus instrument.

### 4.7. Quantum Chemical Calculations

Calculations were performed with the ORCA program, version 4.2.1 [49,50]. The density functional theory-based protocol consisted of the ⍵B97M–D3BJ functional [51,52] (including the D3 dispersion correction by Grimme and coworkers) [53,54] and the triple-zeta basis set def2–TZVPP [55]. The RIJCOSX [56,57] approximation was used to calculate Coulomb and Exchange integrals, using the def2/J auxiliary basis set by Weigend et al. [58] and the GridX5 (ORCA keyword) grid was used. Tight integration grids for the exchange–correlation terms were also used (Grid5, FinalGrid6 keywords in ORCA). Interaction energies were calculated as electronic energies from relaxed structural optimizations in the gas phase.

## Figures and Tables

**Table 1:** Gelation test for **L_1_** and **L_2_** at 1.0 wt% in various solvent/water mixtures (1:1, *v*/*v*).

Solvent	L_1_	L_2_
Water	Gel	Gel
THF/water	Colloidal	Gel
EtOH/water	Colloidal	Gel
MeOH/water	Colloidal	Gel
Acetonitrile/water	Colloidal	Gel
DMF/water	Gel	Gel
DMA/water	Gel	Gel
DMSO/water	Gel	Gel
EG/water †	Gel	Gel *
DME/water †	Gel	Gel *

† 3:7 *v*/*v*; * 1.5 wt%.

**Table 2:** *T_gel_* (°C) comparison of **4–BPU** and **L_1_** at 1.0 wt%**,** and **3–BPU** and **L_2_** at 2.5 wt% in water and various EG/water ratios.

Water: EG	4–BPU *	L_1_ *	3–BPU **	L_2_ **
10:0	98.1	95.2	Crystal	88.2
9:1	85.6	94.5	Crystal	83.6
8:2	81.2	91.7	57.1	79.1
7:3	74.0	91.0	56.4	74.5
6:4	71.6	89.4	Crystal	72.3
5:5	69.5	84.1	Crystal	71.0

* 1.0 wt% and ** 2.5 wt%.

## Supplementary Materials

The following are available online at https://www.mdpi.com/2310-2861/6/4/41/s1, Figure S1: Strain sweep experiments performed on **4–BPU** and **L_1_** gels at 1.0 wt% in water, and **3–BPU** and **L_2_** gels at 2.5 wt% in EG/water (3:7 *v*/*v*) at 25.0 °C and constant frequency of 1.0 Hz, Figure S2: Frequency sweep experiment performed on **3–BPU** and **L_2_** gels at 2.5 wt% in EG/water (3:7 *v*/*v*), at 25.0 °C and a constant strain of 0.05%, Figure S3: Xerogels of **L_1_** obtained from water at 1.0 wt%, Figure S4: Xerogels of **L_1_** obtained from DMSO/water (1:1 *v*/*v*) at 1.0 wt%, Figure S5: Xerogels of **L_2_** obtained from water at 1.0 wt%, Figure S6: Xerogels of **L_2_** obtained from DMSO/water (1:1 *v*/*v*) at 1.0 wt%, Figure S7. Comparison of the XRPD pattern of simulated **L_1_.H_2_O**, as synthesized and the xerogel from EG/water (3:7 *v*/*v*) and water at 1.0 wt%, Figure S8: XRPD comparison of simulated **L_2_****.EG**, **L_2_****.H_2_O**, bulk crystals of **L_2_**, xerogel from EG/water (3:7 *v*/*v*) at 1.2 wt% and water (1.0 wt%), Figure S9: XRPD comparison of simulated **3–BPU.2EG**, **3–BPU**, bulk crystals of **3–BPU** and xerogels obtained from EG/water (3:7 *v*/*v*), Figure S10: Frequency sweep experiments performed at 25.0 °C at a constant strain of 0.02% on **4–BPU** hydrogel at 1.0 wt%, in presence of three equivalents of anions, Figure S11: Frequency sweep experiments performed at 25.0 °C at a constant strain of 0.02% on **L_1_** hydrogel at 1.0 wt%, in presence of three equivalents of anions, Figure S12: Frequency sweep experiments performed at 25.0 °C at a constant strain of 0.02% on **L_2_** hydrogel at 1.0 wt%, in presence of three equivalents of anions, Figure S13: DFT-optimized geometries and calculated interaction energies of various **3–BPU** hydrogen-bonding interactions, Figure S14: DFT-optimized geometries and calculated interaction energies of various **4–BPU** hydrogen-bonding interactions, Figure S15: DFT-optimized geometries and calculated interaction energies of various **L_1_** hydrogen-bonding interactions, Figure S16: DFT-optimized geometries and calculated interaction energies of various **L_2_** hydrogen-bonding interactions. Table S1: Gelation experiment with **4–BPU** and **3–BPU** in water and 1:1 solvent/water mixture, Table S2: Determination of MGC, Table S3: Crystal data, Table S4: Hydrogen-bonding table.
