# Supplementary material for: Role of N–Oxide Moieties in Tuning Supramolecular Gel-State Properties
Source: Gels. 2020 Nov 20;6(4):41. doi: 10.3390/gels6040041 (PMC7709621; doi:10.3390/gels6040041)
Supplement: Supplementary file 1 [file gels-06-00041-s001.pdf]

Supplementary Materials

Role of N–Oxide Moieties in Tuning Supramolecular Gel–State Properties

Dipankar Ghosh, Ragnar Bjornsson and Krishna K. Damodaran*

Table of content

1. Gelation details …………………………………………………………….. 2
2. Rheology ………………………………………………………………… 3
3. Scanning electron microscopy ……… ……………………………………….. 5
4. Crystal data ……………………………………………………………… 7
5. Powder X-ray diffraction ………. …………….…………………………….... 9
6. Stimuli-responsive property …..……………….…………………………….... 12
7. Computational study ………………………………………………………….. 15

1. Gelation details

Table S1: Gelation experiment with **4-BPU** and **3-BPU** in water and 1:1 solvent/water mixture

**Solvent**	**4-BPU** (1.0 wt%)	**3-BPU** (6.0 wt%)
Water	Gel	Insoluble
DMF/water	Gel	Crystal
DMA/water	Gel	Crystal
DMSO/water	Gel	Crystal
MeOH/water	Gel	Crystal
EtOH/water	Gel	Crystal
MeCN/water	Gel	Crystal
THF/water	Gel	Crystal
EG/water	Gel	Gel†
DME/water	Gel	Gel†

†= 3.0 wt%

Table S2: Determination of MGC (wt%)

**Solvent/solvent mixture**	**Water**	**EG/water (3:7 v/v)**
**4-BPU**	0.8*	0.7
**L_1**	0.7	0.7
**3-BPU**	No gelation	2.2
**L_2**	0.8	1.1

* Kumar, D.K.; Jose, D.A.; Das, A.; Dastidar, *Chem. Commun.* **2005**, 4059–4061

2. Rheology

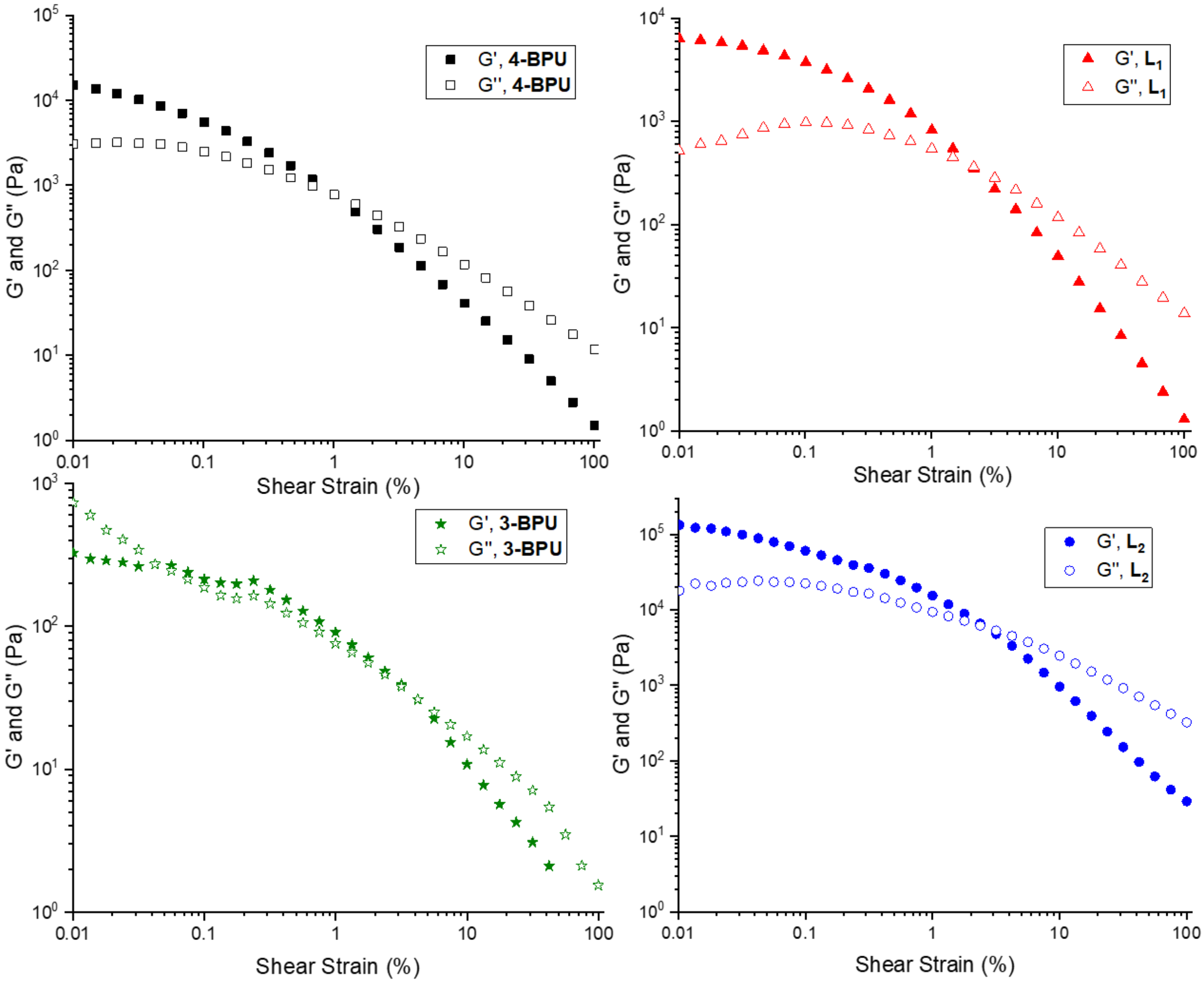

Figure S1: Strain sweep experiments performed on **4-BPU** and $\mathbf{L_1}$ gels at 1.0 wt% in water, and **3-BPU** and $\mathbf{L_2}$ gels at 2.5 wt% in EG/water (3:7 v/v) at 25.0 °C and constant frequency of 1.0 Hz.

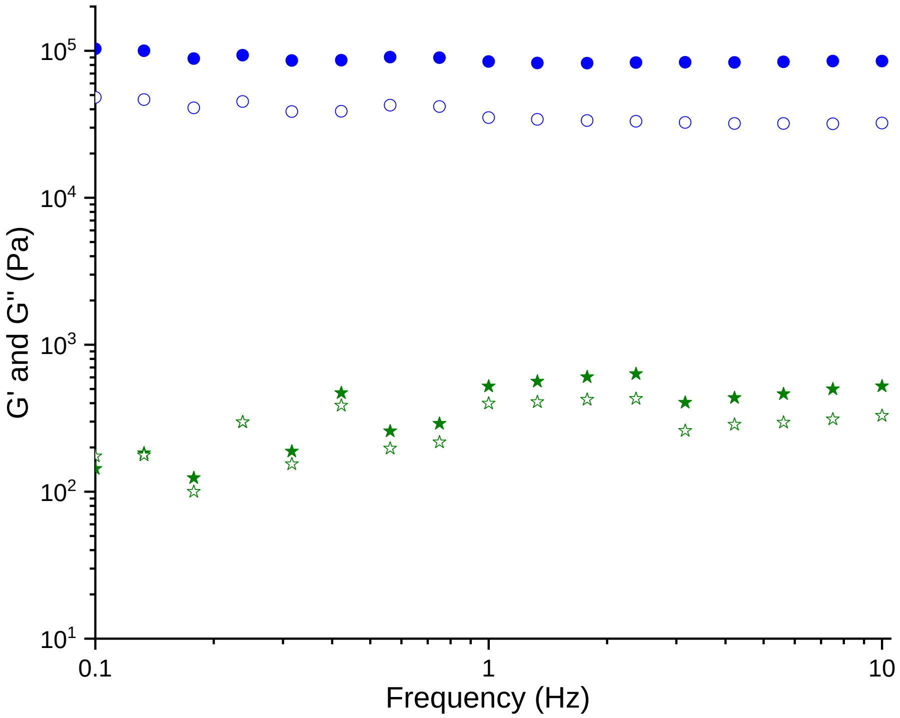

Figure S2: Frequency sweep experiment performed on **3-BPU** and **L_2** gels at 2.5 wt% in EG/water (3:7 v/v), at 25.0 °C and a constant strain of 0.05%. Colour codes: G’, **3-BPU** (★), G’’, **3-BPU** (☆), G’, **L_2** (●) and G’’, **L_2** (○).

3. Scanning electron microscopy

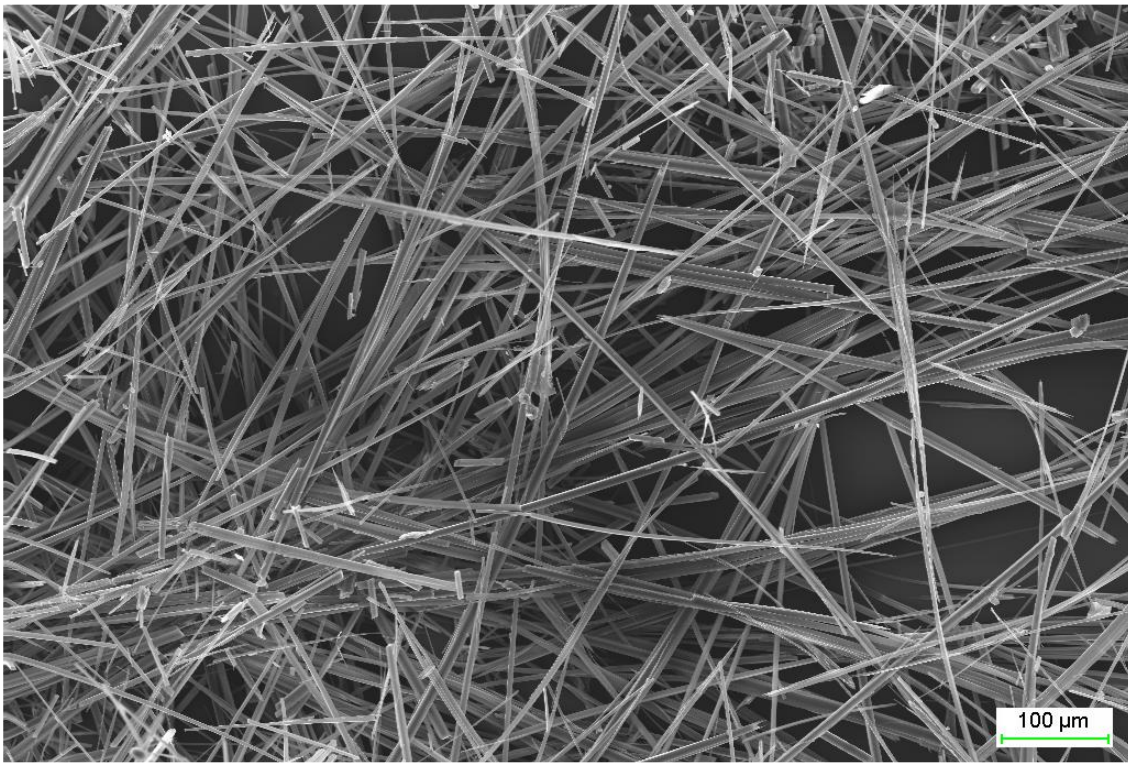

Figure S3: Xerogels of $\mathbf{L_1}$ obtained from water at 1.0 wt%.

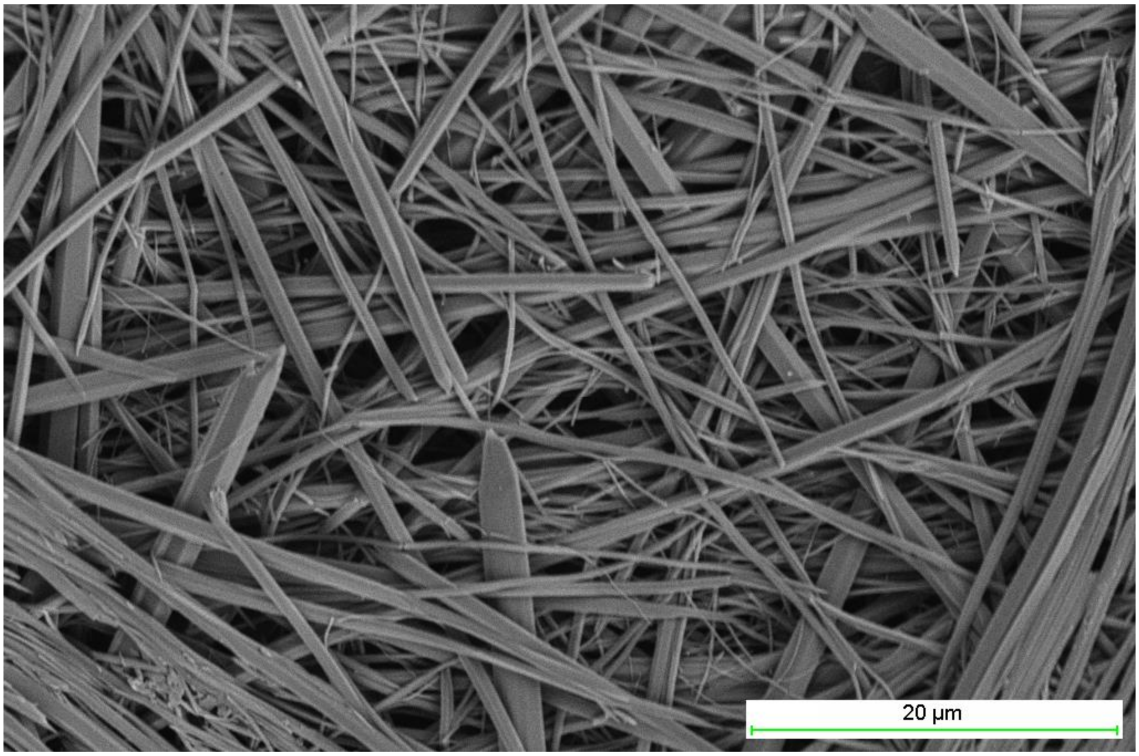

Figure S4: Xerogels of $\mathbf{L_1}$ obtained from DMSO/water (1:1 v/v) at 1.0 wt%.

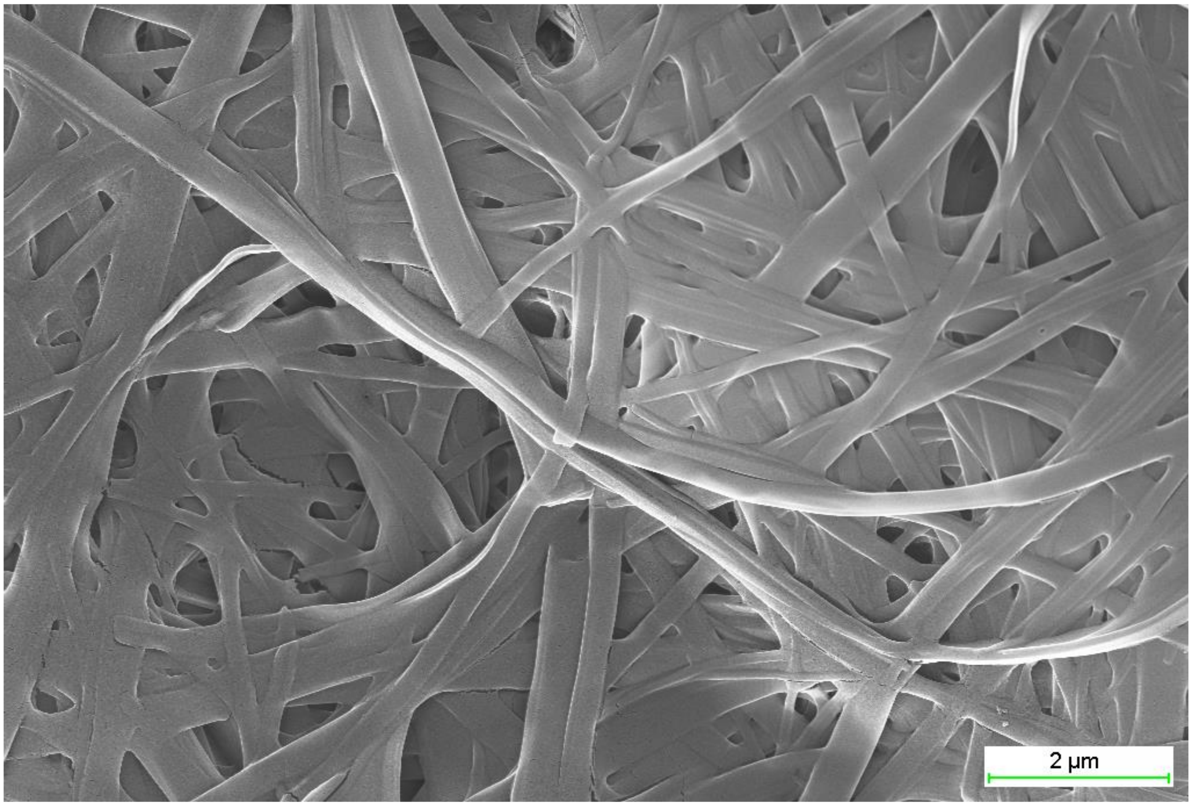

Figure S5: Xerogels of $\mathbf{L_2}$ obtained from water at 1.0 wt%.

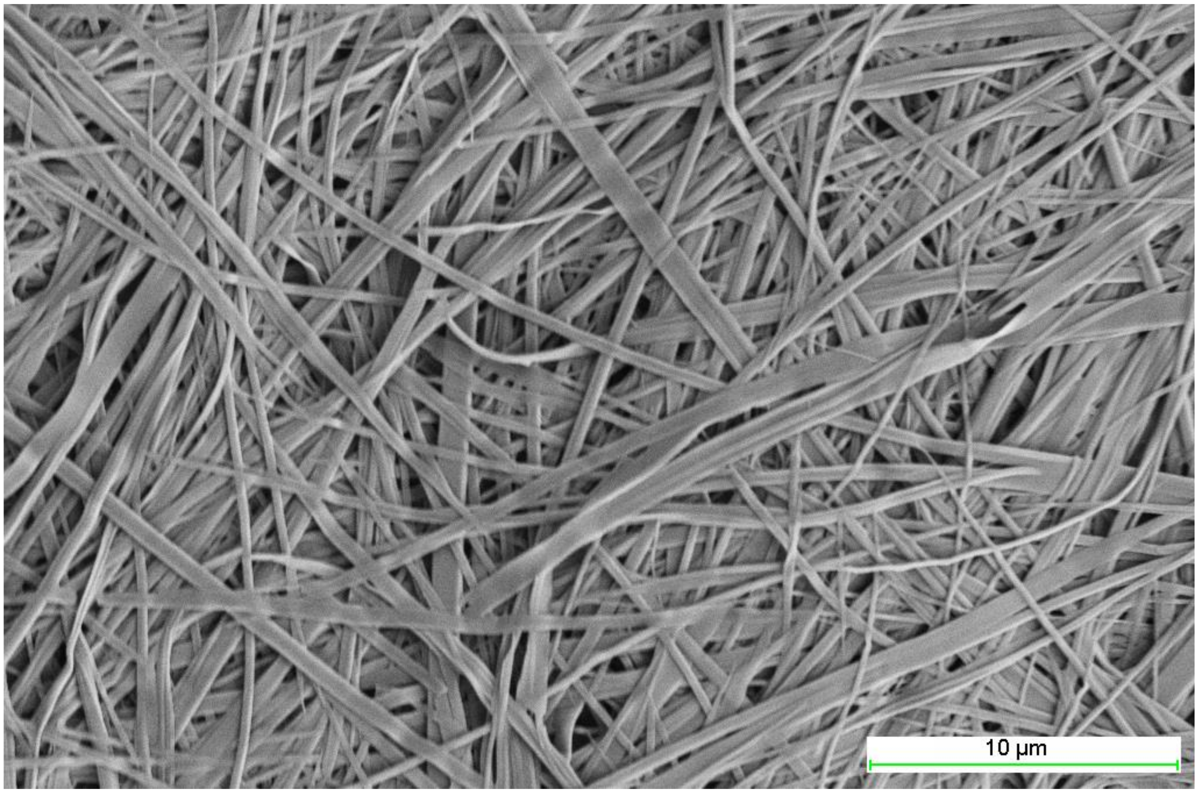

Figure S6: Xerogels of $\mathbf{L_2}$ obtained from DMSO/water (1:1 v/v) at 1.0 wt%.

4. Crystal data

Table S3: Crystal data

Crystal data	**$L_1.H_2O$**	**$L_2.EG$**	**3-BPU.EG**
Empirical formula	$C_{11}H_{12}N_4O_4$	$C_{13}H_{16}N_4O_5$	$C_{14}H_{19}N_4O_4$
Colour	Colourless	Colourless	Colourless
Formula weight	264.25	308.30	307.33
Crystal size (mm)	0.23×0.05×0.04	0.28×0.1×0.05	0.42×0.1×0.075
Crystal system	monoclinic	orthorhombic	monoclinic
Space group	$P2_1/c$	$C222_1$	$P2_1/c$
a (Å)	3.79670(10)	7.8647(4)	18.3475(7)
b (Å)	12.2604(3)	11.0925(5)	4.7351(2)
c (Å)	24.6800(5)	15.9892(8)	17.5516(6)
α (°)	90	90	90
β (°)	90.3160(10)	90	98.4340(10)
γ (°)	90	90	90
Volume ($Å^3$)	1148.81(5)	1394.88(12)	1508.34(10)
Z	4	4	4
$D_{calc.}$ (g/cm^3)	1.528	1.468	1.353
F(000)	552	648	652
μ CuKα (mm^{-1})	1.011	0.972	0.843
Temperature (K)	150(2)	150(2)	150(2)
Reflections collected/ unique/observed [I>2σ(I)]	13589/2026/ 1794	4732/1366/1334	23481/2950/2768
Data/restraints/parameters	2026/0/180	1366/0/103	2950/0/223
Goodness of fit on F^2	1.045	1.138	1.017
Final R indices [I>2σ(I)]	R_1 = 0.0350<br>wR_2 = 0.0817	R_1 = 0.0296<br>wR_2 = 0.0853	R_1 = 0.0370<br>wR_2 = 0.0957
R indices (all data)	R_1 = 0.0411<br>wR_2 = 0.0855	R_1 = 0.0302<br>wR_2 = 0.0858	R_1 = 0.0389<br>wR_2 = 0.0972

Table S4: Hydrogen-bonding table

**$L_1.H_2O$**						
Nr	Donor---H···Acceptor	D–H/**Å**	H···A/**Å**	D···A/**Å**	∠D---H···A/°	Symmetry operation
1	N(8)---H(8)···O(16)	0.88	1.98	2.7990(16)	153	1-x,1/2+y,3/2-z
2	N(11)---H(11)···O(16)	0.88	1.96	2.7851(16)	156	1-x,1/2+y,3/2-z
3	O(19)---H(19A)···O(1)	0.96(3)	1.80(3)	2.7466(19)	172(2)	x,y,z
4	O(19)---H(19A)···N(2)	0.96(3)	2.50(3)	3.2906(19)	141(2)	x,y,z
5	O(19)---H(19B)···O(1)	0.90(2)	1.87(2)	2.7612(19)	168(2)	-1+x,y,z
6	C(3)---H(3)···O(10)	0.95	2.53	3.2099(18)	128	1-x,1-y,1-z
7	C(6)---H(6)···O(19)	0.95	2.46	3.280(2)	144	2-x,2-y,1-z
8	C(17)---H(17)···O(19)	0.95	2.59	3.415(2)	146	-1+x,3/2-y,1/2+z
**L_2.EG**						
Nr	Donor---H···Acceptor	D–H/**Å**	H···A/**Å**	D···A/**Å**	∠D---H···A/°	Symmetry operation
1	N(8)---H(8)···O(1)	0.88	2.07	2.690(2)	127	1/2+x,-1/2+y,z
2	O(11)---H(11)···O(1)	0.77(3)	1.97(3)	2.741(2)	175.1(17)	x,y,z
3	C(3)---H(3)···O(11)	0.95	2.45	3.377(3)	164	-1/2+x,3/2-y,1-z
4	C(4)---H(4)···O(11)	0.95	2.52	3.208(3)	129	-1/2+x,-1/2+y,z
**3-BPU.EG**						
Nr	Donor---H···Acceptor	D–H/**Å**	H···A/**Å**	D···A/**Å**	∠D---H···A/°	Symmetry operation
1	N(7)---H(7)···O(17)	0.88	1.92	2.7496(13)	158	x,1/2-y,1/2+z
2	N(10)---H(10)···O(20)	0.88	2.13	2.9122(14)	147	x,1/2-y,1/2+z
3	O(17)---H(17)···O(21)	0.84	1.88	2.7026(15)	166	x,y,z
4	O(20)---H(20)···N(13)	0.84	1.97	2.7932(15)	166	1-x,-1/2+y,3/2-z
5	O(21)---H(21)···N(1)	0.908(19)	1.838(19)	2.7344(14)	168.6(17)	x,y,z

5. Powder X-ray diffraction

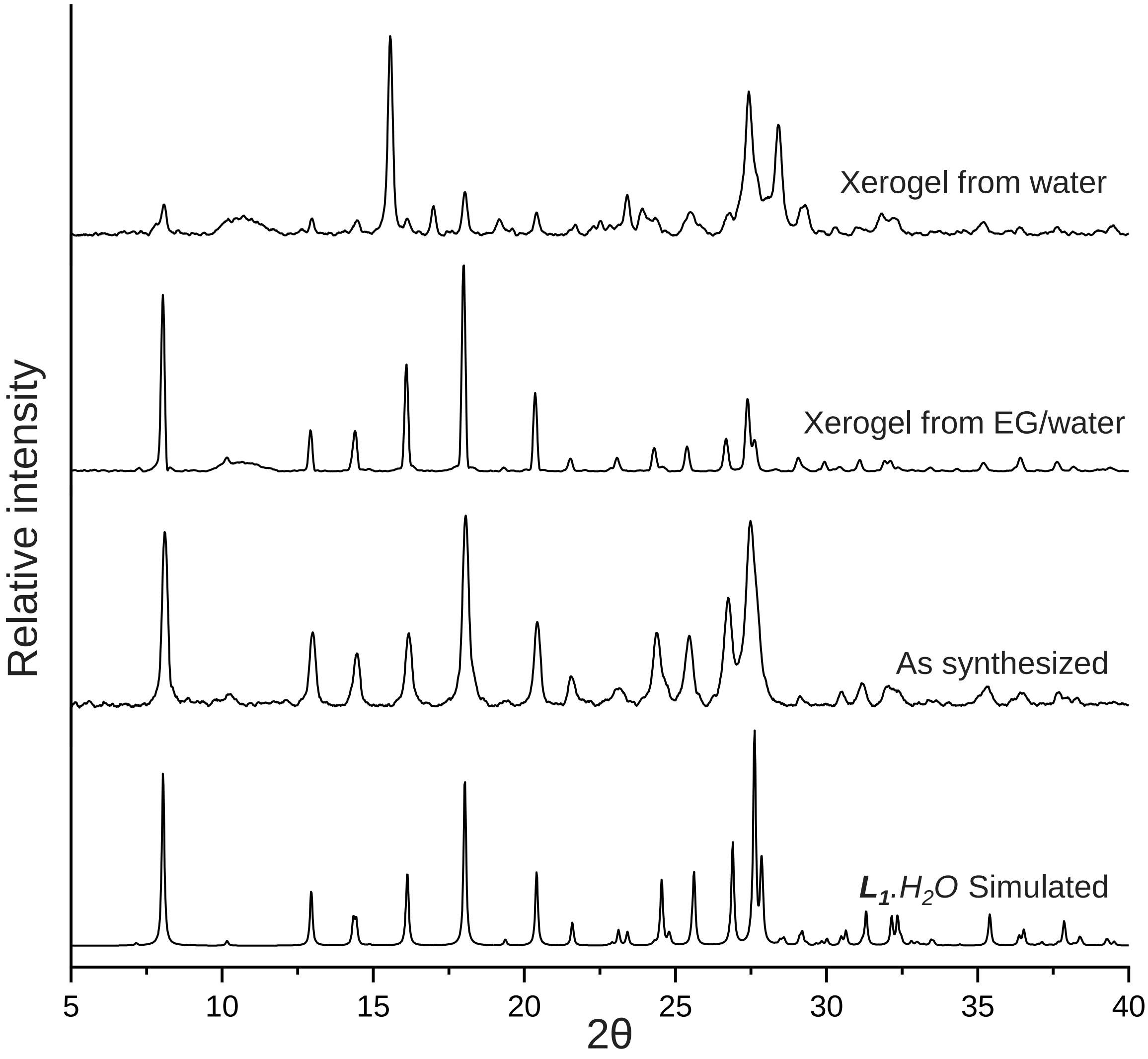

Figure S7: Comparison of the XRPD pattern of simulated **$L_1.H_2O$**, as synthesized and the xerogel from EG/water (3:7 v/v) and water at 1.0 wt%.

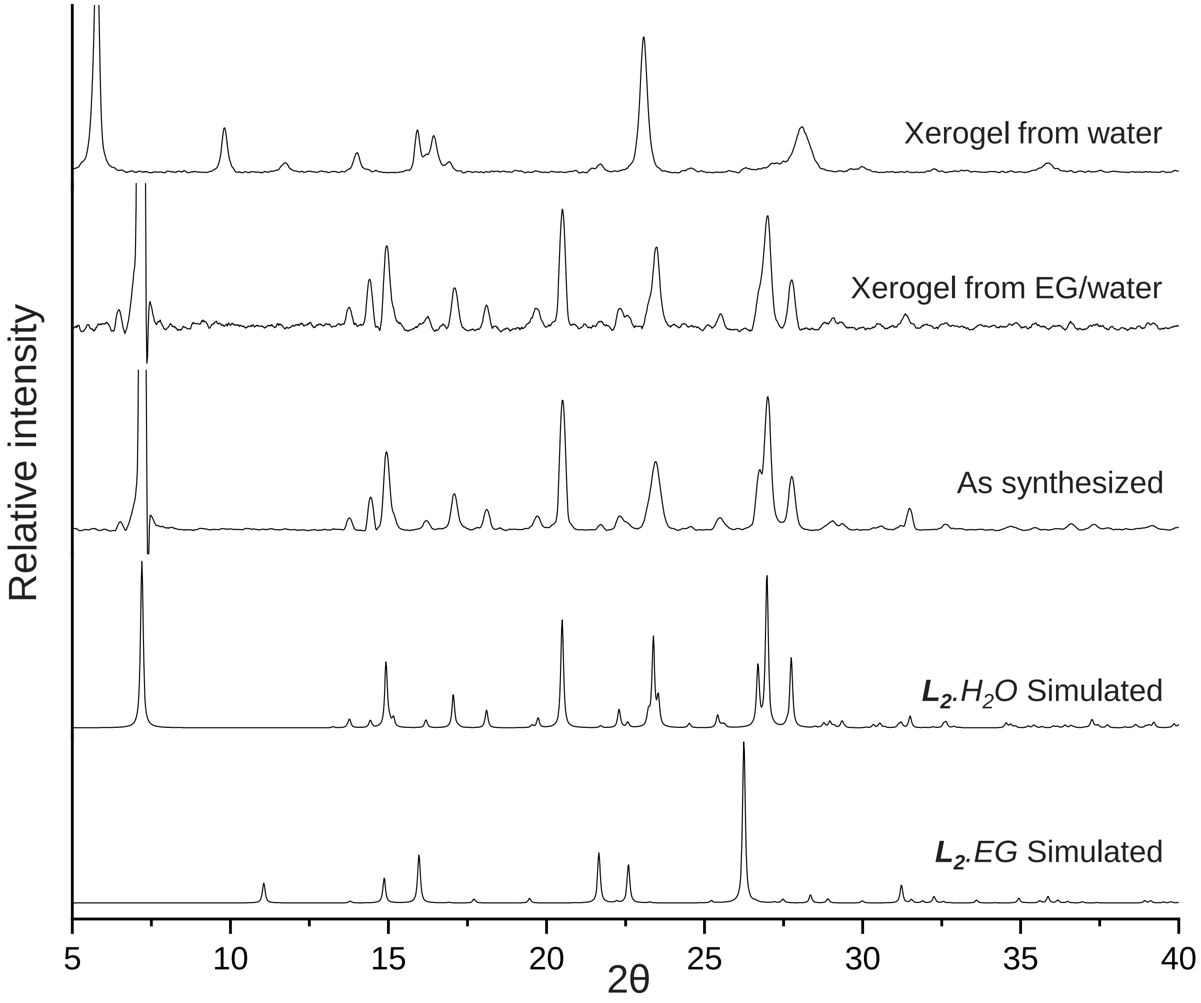

Figure S8: XRPD comparison of simulated **L_2.EG**, **L_2.H_2O**, bulk crystals of **L_2**, xerogel from EG/water (3:7 v/v) at 1.2 wt% and water (1.0 wt%).

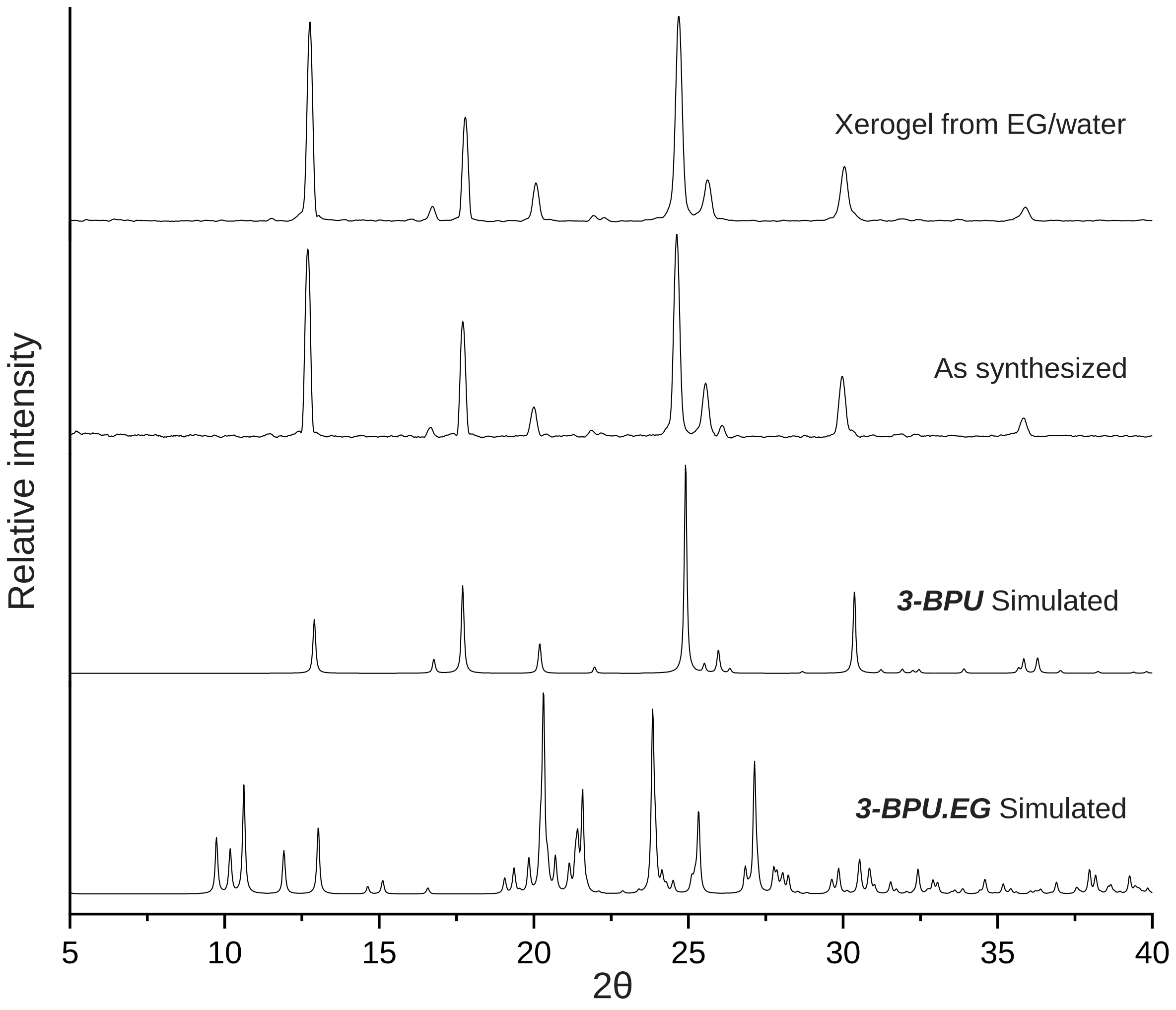

Figure S9: XRPD comparison of simulated **3-BPU.2EG**, **3-BPU**, bulk crystals of **3-BPU** and xerogels obtained from EG/water (3:7 v/v).

6. Stimuli-responsive property

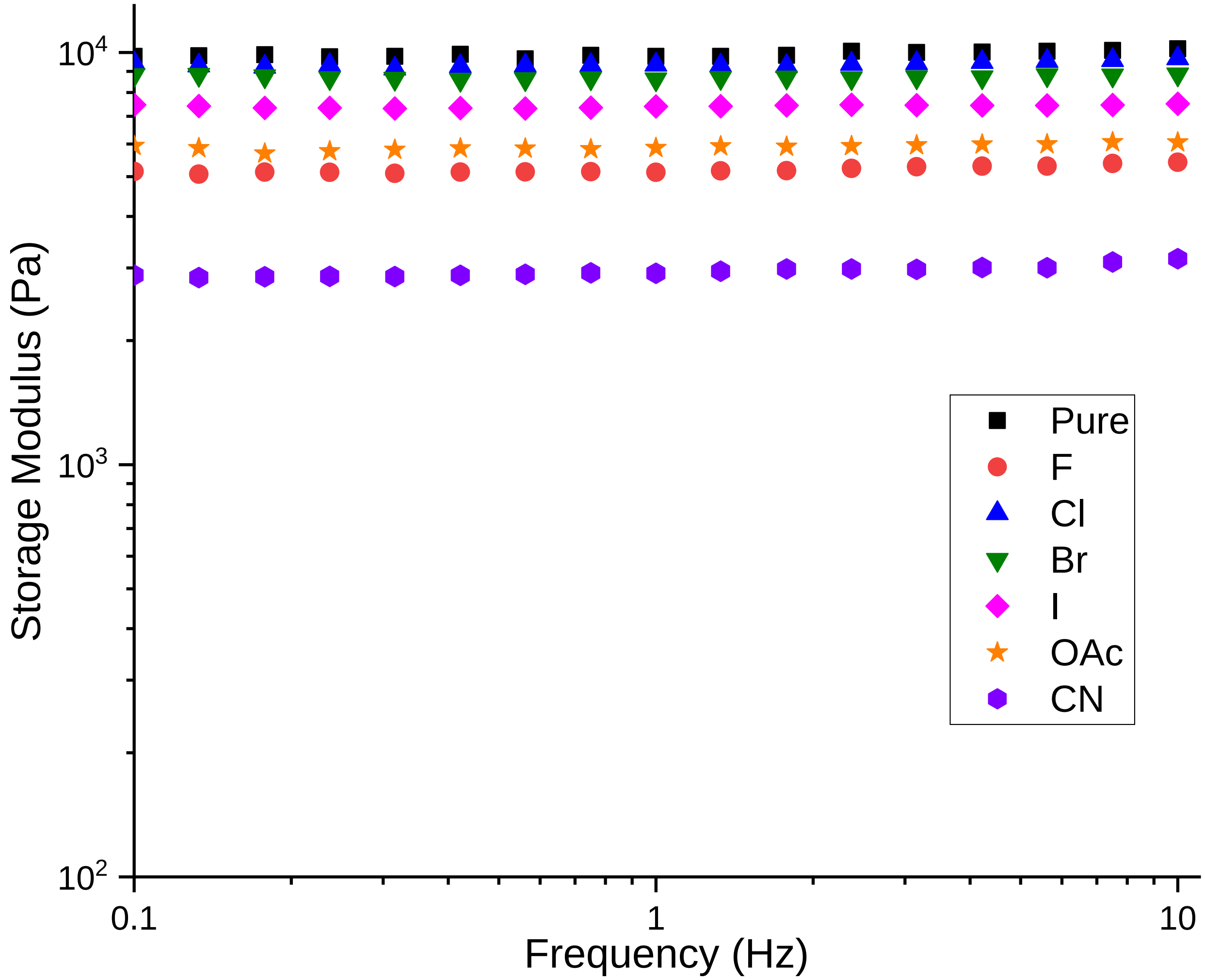

Figure S10: Frequency sweep experiments performed at 25.0 °C at a constant strain of 0.02% on **4-BPU** hydrogel at 1.0 wt%, in presence of three equivalents of anions.

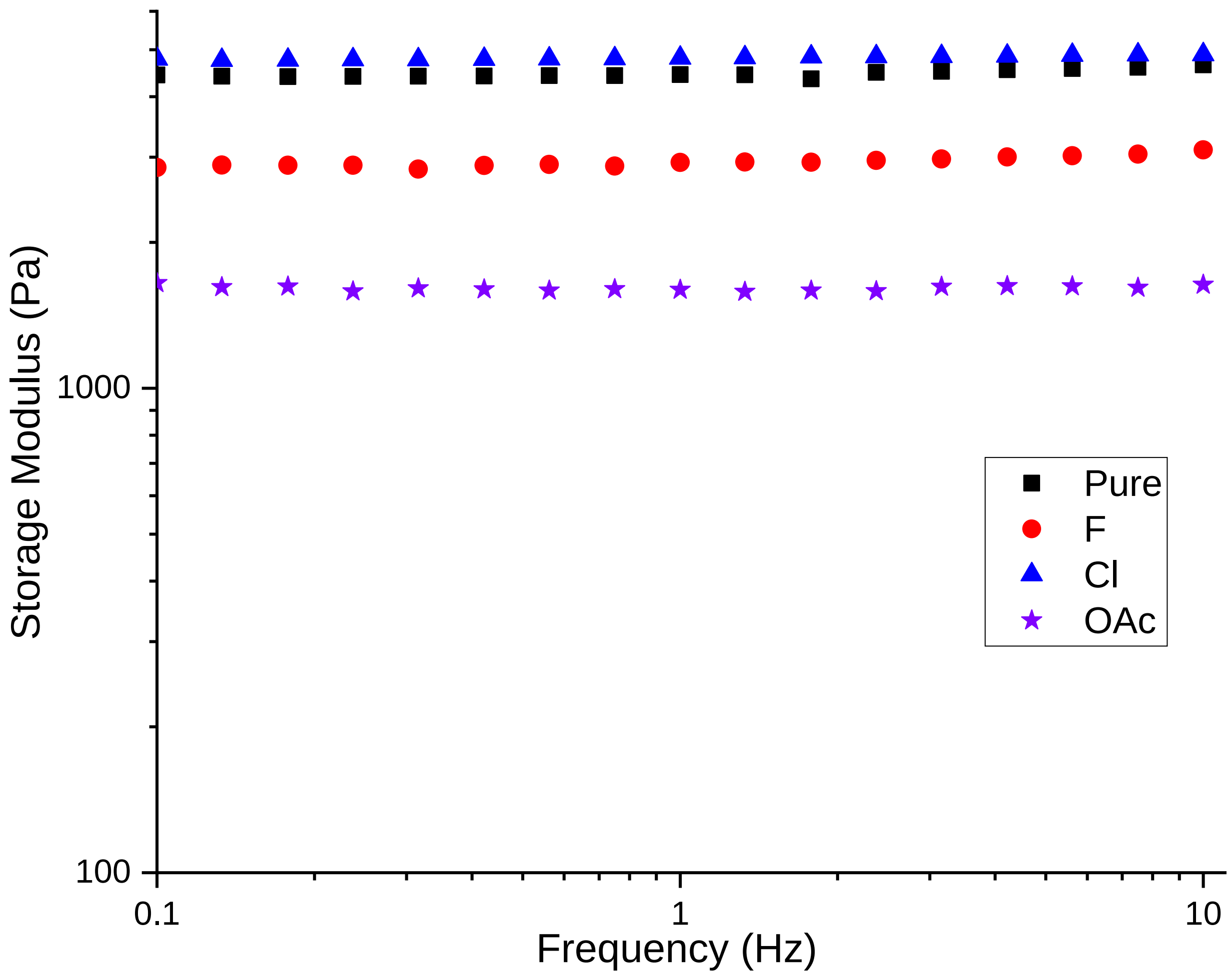

Figure S11: Frequency sweep experiments performed at 25.0 °C at a constant strain of 0.02% on $\mathbf{L_1}$ hydrogel at 1.0 wt%, in presence of three equivalents of anions.

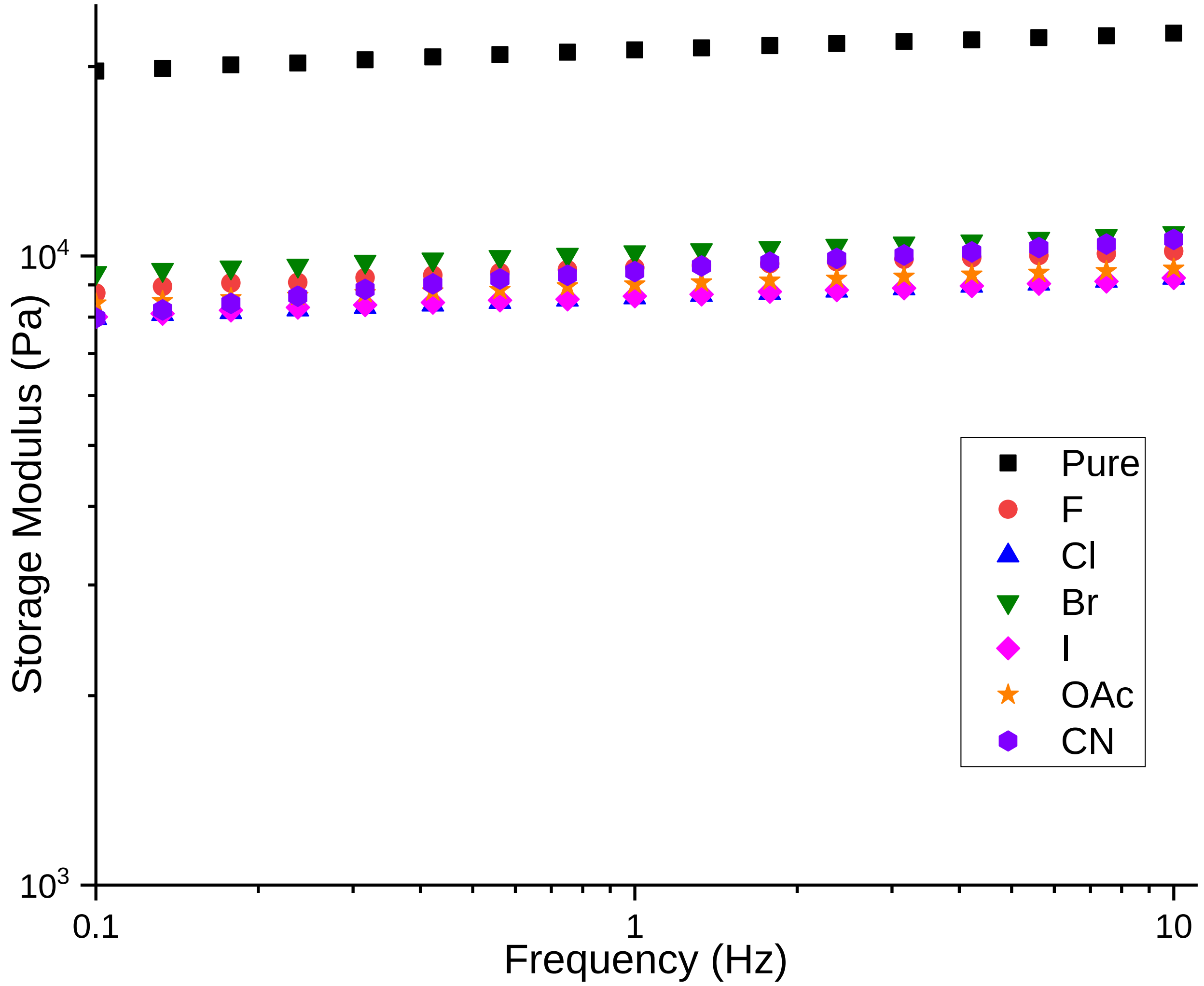

Figure S12: Frequency sweep experiments performed at 25.0 °C at a constant strain of 0.02% on $\mathbf{L_2}$ hydrogel at 1.0 wt%, in presence of three equivalents of anions.

7. Computational study

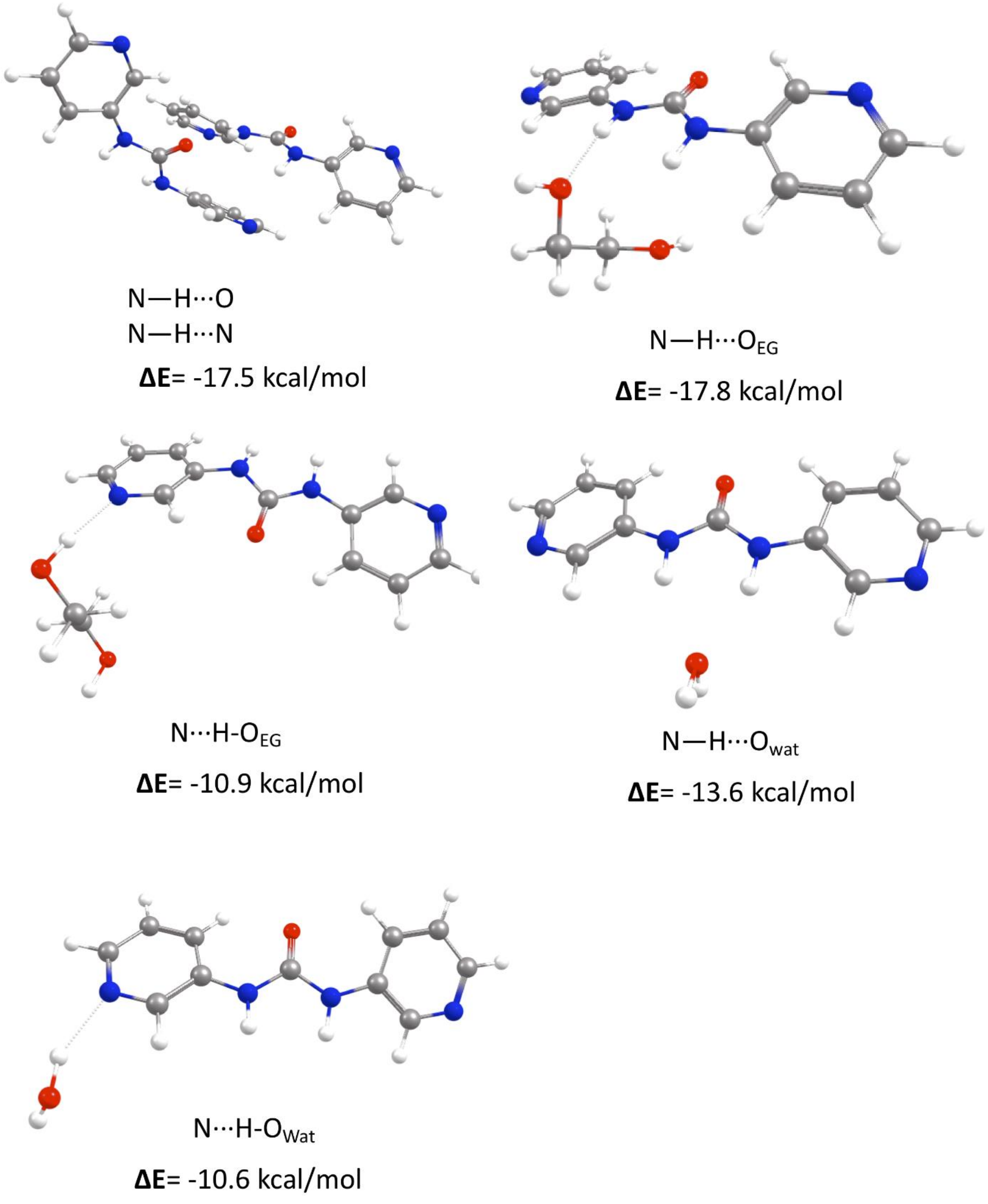

Figure S13: DFT-optimized geometries and calculated interaction energies of various **3-BPU** hydrogen-bonding interactions.

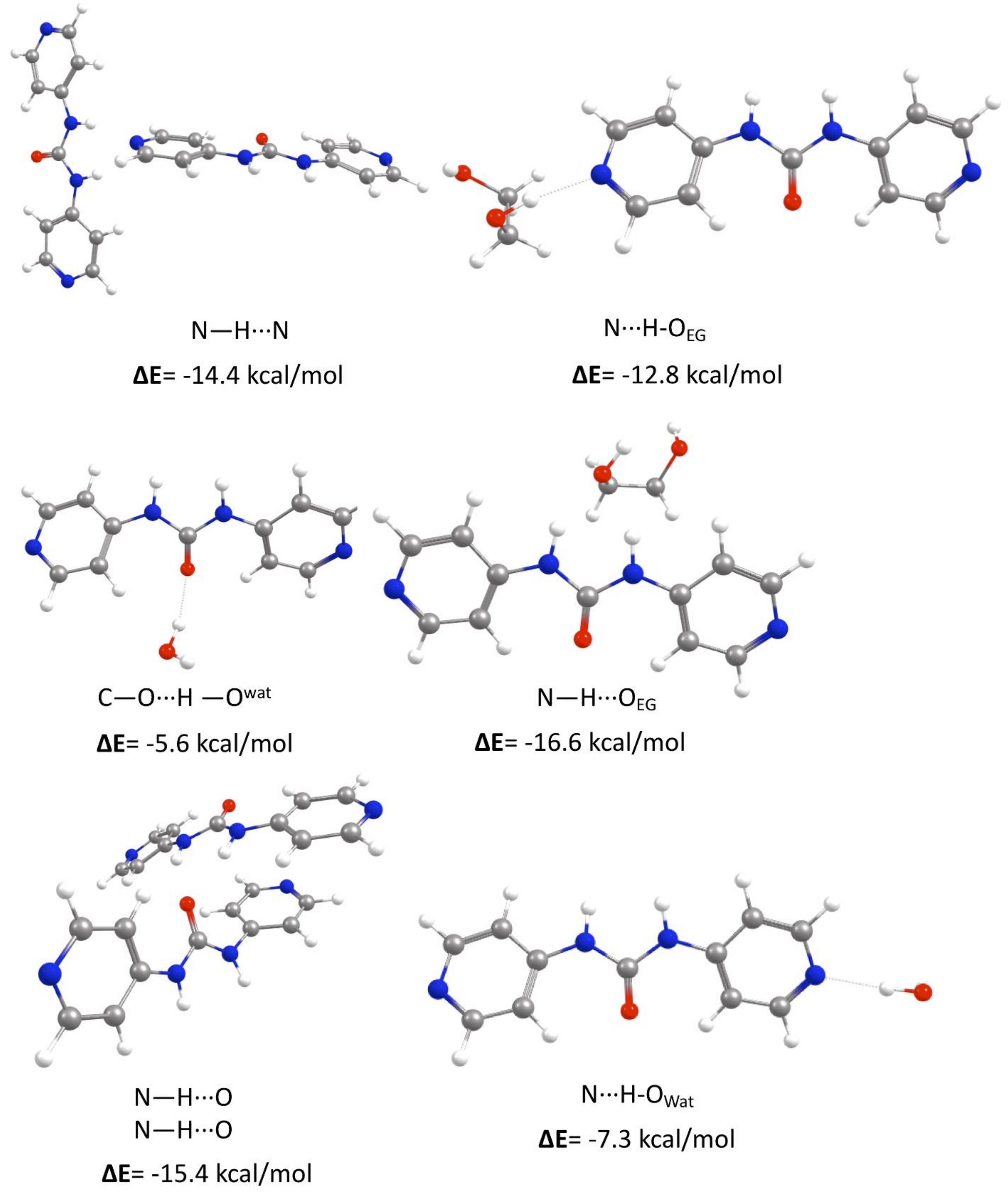

Figure S14: DFT-optimized geometries and calculated interaction energies of various **4-BPU** hydrogen-bonding interactions.

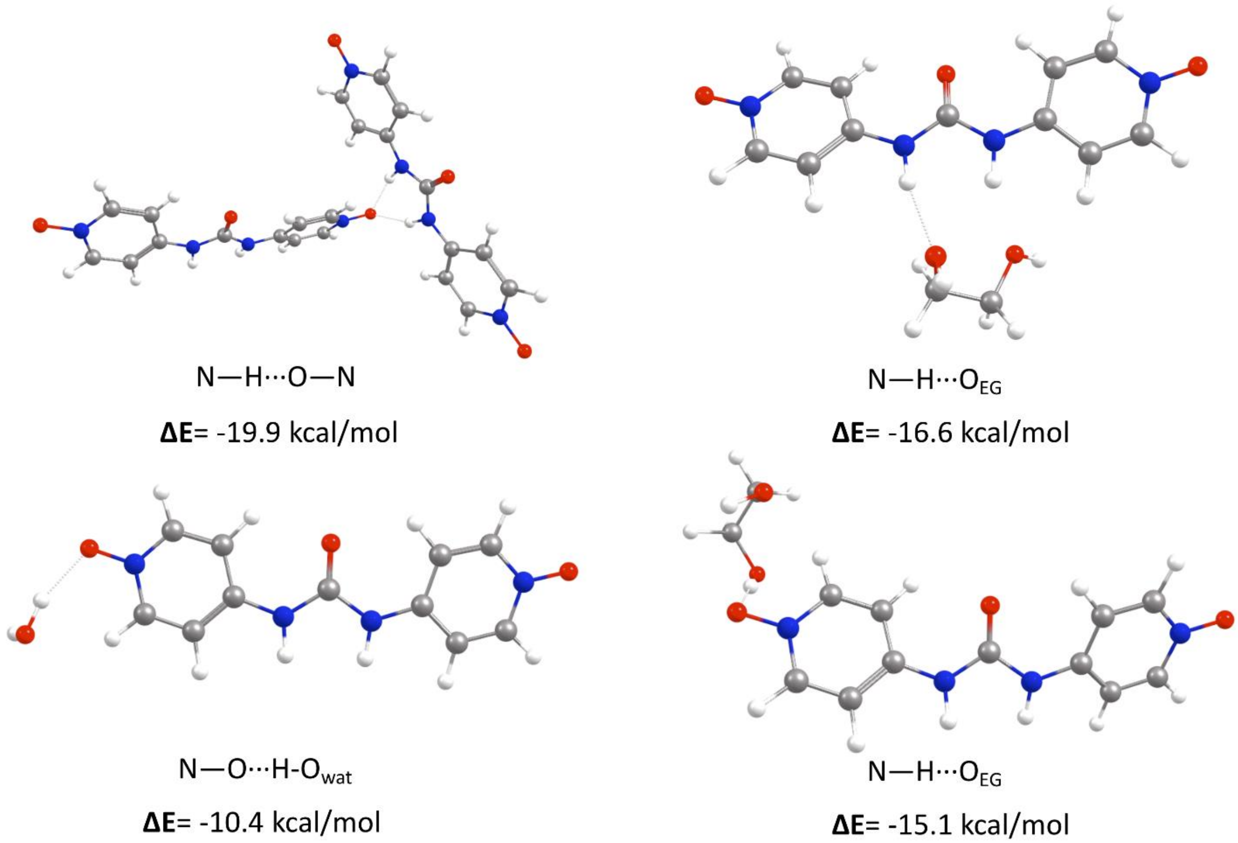

Figure S15: DFT-optimized geometries and calculated interaction energies of various $\mathbf{L_1}$ hydrogen-bonding interactions.

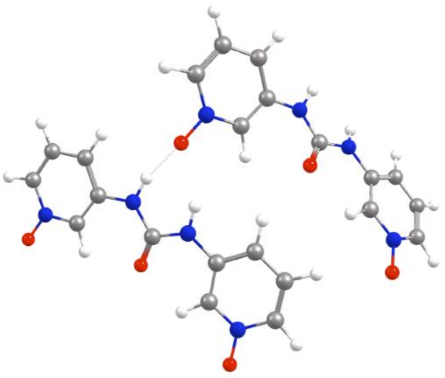

N—H···O—N

ΔE= -19.6 kcal/mol

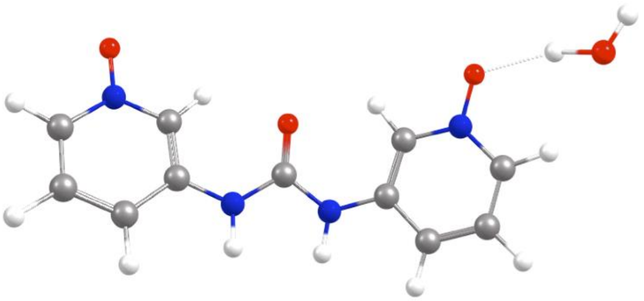

N—O···H-O_{wat}

ΔE= -8.8 kcal/mol

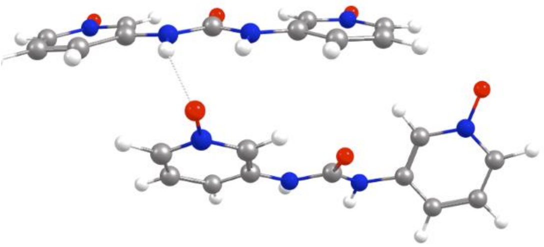

N—H···O-N

ΔE= -24.3 kcal/mol

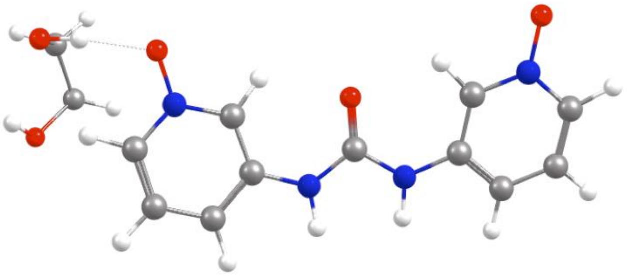

N—O···H-O_{EG}

ΔE= -15.1 kcal/mol

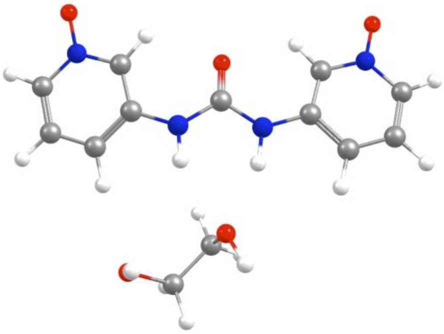

N—H···O_{EG}

ΔE= -15.0 kcal/mol

Figure S16: DFT-optimized geometries and calculated interaction energies of various $\mathbf{L_2}$ hydrogen-bonding interactions.
